# Quantitative matching of forensic evidence fragments utilizing 3D microscopy analysis of fracture surface replicas

**DOI:** 10.1111/1556-4029.15012

**Published:** 2022-03-07

**Authors:** Bishoy Dawood, Carlos Llosa‐Vite, Geoffrey Z. Thompson, Barbara K. Lograsso, Lauren K. Claytor, John Vanderkolk, William Meeker, Ranjan Maitra, Ashraf Bastawros

**Affiliations:** ^1^ Department of Aerospace Engineering Iowa State University Ames Iowa USA; ^2^ Department of Statistics Iowa State University Ames Iowa USA; ^3^ Department of Mechanical Engineering Iowa State University Ames Iowa USA; ^4^ Virginia Department of Forensic Science Richmond Virginia USA; ^5^ Retired from Indiana State Police Laboratory Fort Wayne Indiana USA

**Keywords:** cast surface replica, fracture match, microscopic surface characterization, physical match, statistical and classification model, surface topography comparison, trace evidence

## Abstract

Silicone casts are widely used by practitioners in the comparative analysis of forensic items. Fractured surfaces carry unique details that can provide accurate quantitative comparisons of forensic fragments. In this study, a statistical analysis comparison protocol was applied to a set of 3D topological images of fractured surface pairs and their replicas to provide confidence in the quantitative statistical comparison between fractured items and their silicone cast replicas. A set of 10 fractured stainless steel samples were fractured from the same metal rod under controlled conditions and were replicated using a standard forensic casting technique. Six 3D topological maps with 50% overlap were acquired for each fractured pair. Spectral analyses were utilized to identify the correlation between topological surface features at different length scales of the surface topology. We selected two frequency bands over the critical wavelength (greater than two‐grain diameters) for statistical comparison. Our statistical model utilized a matrix‐variate *t*‐distribution that accounts for overlap between images to model match and non‐match population densities. A decision rule identified the probability of matched and unmatched pairs of surfaces. The proposed methodology correctly classified the fractured steel surfaces and their replicas with a posterior probability of match exceeding 99.96%. Moreover, the replication technique shows potential in accurately replicating fracture surface topological details with a wavelength greater than 20 μm, which far exceeds the feature comparison range on most metallic alloy surfaces. Our framework establishes the basis and limits for forensic comparison of fractured articles and their replicas while providing a reliable fracture mechanics‐based quantitative statistical forensic comparison.

**Highlights**

- Silicone casts are widely used in the comparative analysis of forensic items.
- 3D topological images of fractured surface pairs and their replicas are acquired.
- Statistical learning is used to compare fracture items and their replicas.
- Silicon casts accurately replicate fracture surface topological details of 20  μm wavelength or larger.

## 1 INTRODUCTION

A physical fit or physical match, as described by the American Society of Trace Evidence Examiners (ASTEE), is the alignment between two or more pieces to determine whether they once formed a single object [1, 2]. Matching the physical fractures of different materials such as wood, glass, paper, skin, cables, tapes, and metals has been widely studied [3, 4, 5, 6, 7, 8, 9, 10, 11, 12, 13, 14, 15, 16, 17, 18]. This has also been extended to the examination of tool marks on human tissues (e.g., bone and cartilage) [19, 20, 21, 22, 23, 24]. This physical matching utilizes the thickness, color, pattern, fracture morphology, irregularities in the fracture, and imperfections across the fracture location [25, 26]. Patterns along with the complex jagged trajectory of a macro‐crack (large cracks visible to the naked eye) are considered unique and can be utilized to distinguish matching pairs of fractured surfaces by an examiner or by a layperson on a jury [20, 27, 28]. However, reliable examination decisions require experienced forensic experts using comparative microscopy and physical pattern matching. Moreover, the error rate is difficult to quantify in physical matching due to many factors, including fragment material properties, loading and environmental exposure, and forensic scientist judgment and experience [2, 19, 20].

**FIGURE 1:**
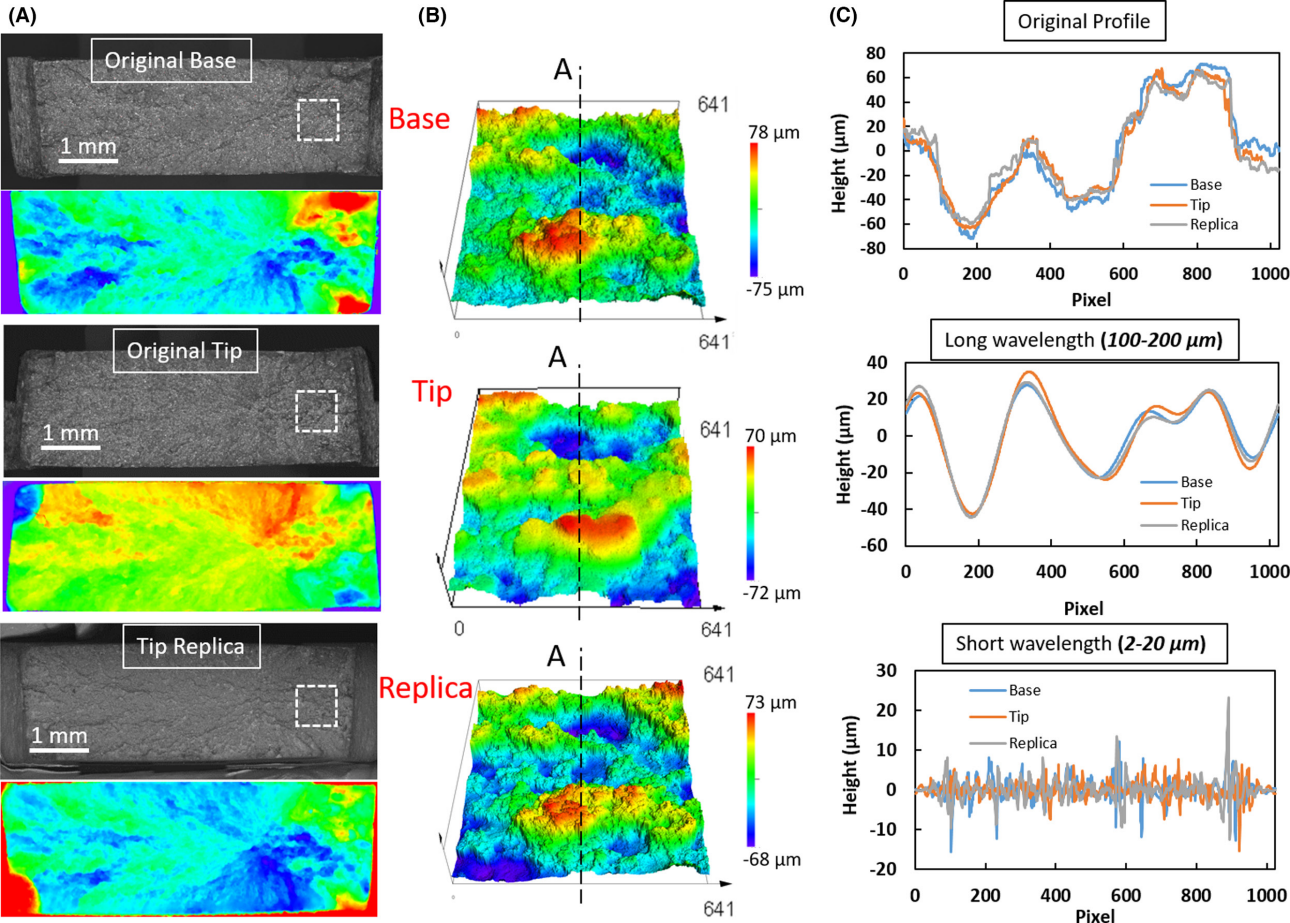
3D topological analysis of a Base‐Tip pair of fractured surfaces and a Tip‐cast replica. (A) Optical micrograph and color rendering of the topological fracture surface. Base/Tip and tip/its cast show mirror symmetry. (B) 3D topological representation of the fracture surface, utilizing a 640 μm field of view for the square inset area on the optical image. (C) 2D representation of the height comparison along with line A–A showing the original measured height (top) and the corresponding long‐wavelength (middle) and short‐wavelength (bottom), utilizing spectral decomposition by Fourier transform analysis. The low‐frequency topological details (middle) exhibit patterns that are relevant for statistical comparison while the short‐wavelengths topological details (bottom) exhibit no comparable patterns

During the last two decades, new innovative 3D surface topological scanning microscopy has been developed with the potential to improve physical matching. Different 3D acquisition systems, employing 3D laser scanners, optical coherence tomography, stylus scanning instruments, and confocal microscopy, have been utilized for forensic evidence identification applications [19, 26, 29, 30, 31, 32, 33, 34, 35, 36, 37, 38, 39, 40, 41, 42, 43]. Automated surface acquisition and matching processes utilizing 3D topography data have demonstrated promising improvements in the objectivity of the comparison process [34]. Specifically, confocal microscopy utilizes a pinhole aperture, allowing only light that is reflected from the in‐focus plane to where it is captured. Confocal microscopy allows for slices of surfaces to be captured at different depths and then stacked on top of each other to render a 3D image of the surface topology. This 3D image can then be visualized as a two‐dimensional profile of the surface roughness, as shown in Figure 1C. Note that we coded the fractured pair surfaces as a “Base” and a “Tip” pair. Zooming out from the profile (looking at longer wavelengths, or spacing between topological events) makes the profile appear smoother, while zooming in on the profile (looking at shorter wavelengths, or spacing between events) results in the 2D profile appear rougher and makes it possible to identify unique features for forensic comparison. Further, forensic replicas utilizing silicone casts are widely used by practitioners in the comparative analysis of physical forensic articles [18, 42, 44, 45] and for tool marks on human tissues [19, 20, 21, 22, 23, 24]. However, there is a dearth of in‐depth analysis of the ability of a silicon cast to reproduce a useful range of topographical details needed for 3D analysis [19]. Especially, the silicon cast of a fracture surface may not replicate all the small features that represent the short‐wavelength topology of the original surface.

**FIGURE 2:**
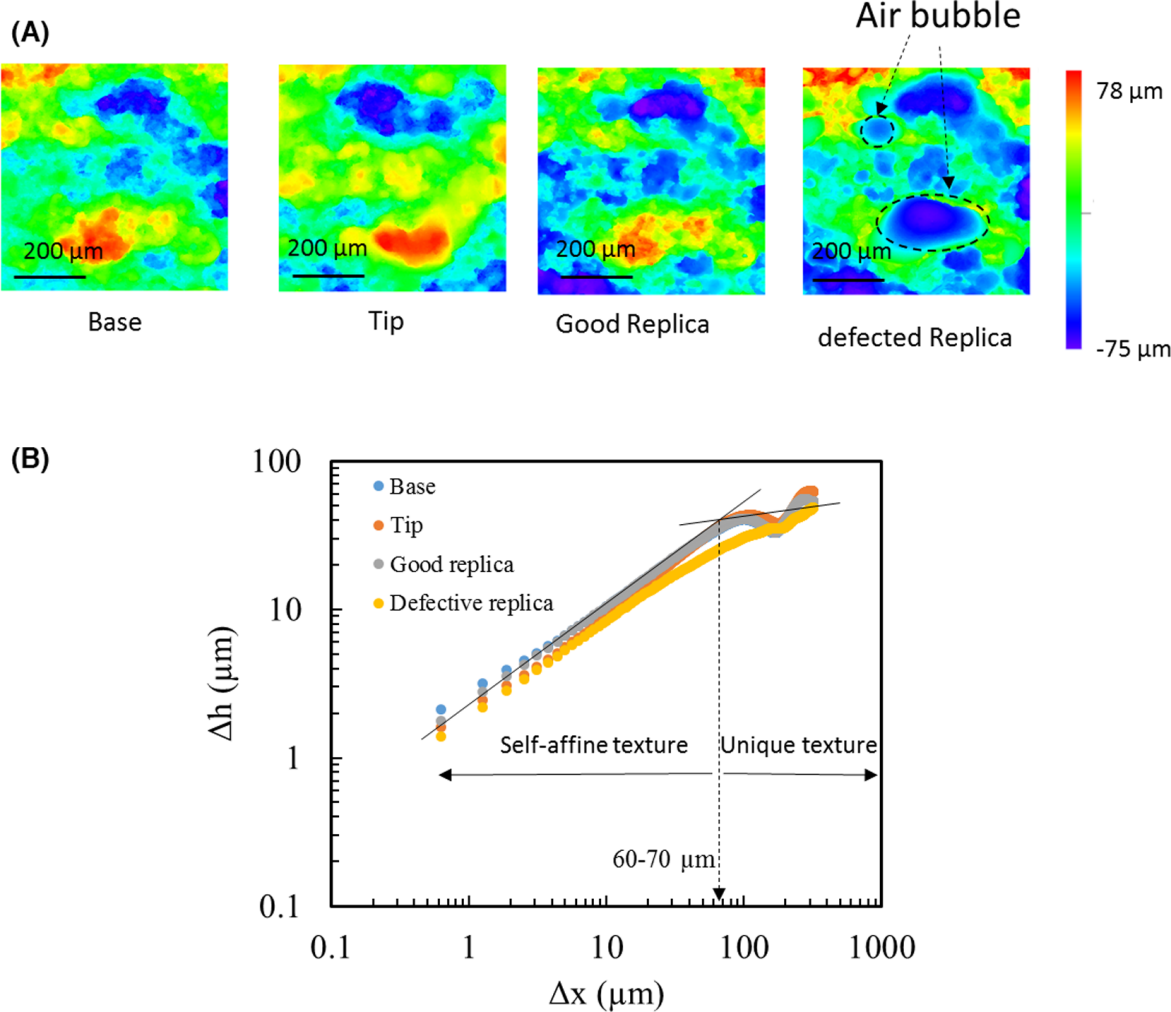
Role of replica quality in identifying unique length scales for fracture comparison. (A) Color map rendering for topological heights, showing a pair of Base‐Tip and good versus defective replicas of the Tip due to large voids. Notice that both the base and the Tip‐replica images are mirror images of the Tip image. (B) The corresponding variation of the height‐height statistical correlation with the size of the imaging window. The existence of a bubble within the field of view altered the critical wavelength at which the topological details show a transition from self‐affine textures to the unique texture that can be used for matching purposes

In this paper, we address the limits and applicability of surface replica for forensic comparison using our formal quantitative framework to quantify the probability of a match between two specimens in question [46, 47]. We combine fracture mechanics with statistics and machine learning to arrive at a comparison decision. When the 3D spectral analysis of the fractured surface topography is combined with a statistical learning tool, the domain of unique individuality can be easily identified [47]. This tool can provide a quantitative analysis for match probability and the corresponding error rate that is required to be reported [48, 49]. The indefinite microscopic features on the fracture surface topology, as highlighted in Figure 1A,B, carry considerable unique details that may be used to support the forensic examiner’s decisions with a quantitative forensic comparison. The fractured surfaces show self‐affine scaling properties (proportionality of surface roughness with the observation window scale) to quantitatively correlate the material resistance to fracture with the resulting surface roughness, as highlighted in Figure 2. The corresponding surface roughness analysis of this surface is performed by the height‐height correlation function, $\Delta h(\Delta x)=\sqrt{{\left\langle (h(x+\Delta x)-h(x))\right\rangle }_{x}}$, where ${\left\langle \right\rangle }_{x}$ imply averaging over the *x*‐direction. The limit of the self‐affine scale is controlled by the material resistance to fracture, crystal structure, material impurities and loading conditions [12, 50, 51, 52, 53, 54]. The surface characteristic becomes unique and non‐self‐affine at a larger scale, say, λ [54, 55, 56], where it is eclipsed by the interference of the fracture process zone length scale. The observed saturation scale sets two important length scales for the comparison process. These are; (i) the proper imaging scale and the corresponding field of view (FOV) required for proper imaging of the fracture surface topology. This imaging scale should be greater than 10‐times the self‐affine saturation scale. (ii) The proper scale for forensic comparison of fractured surfaces and the corresponding radial comparison bands on the frequency spectral space. Figure 2 shows the fracture surface topology and the corresponding height‐height correlation as a function of the analysis window size. At a larger length scale (λ ~ 60–70 μm for the examined SS alloy), the individuality of the fracture surface topology can exist [36, 47, 53], noted by the saturation of the correlation coefficient. This scale is about the size of the fracture process zone ahead of the crack tip, typically extending to about 2–3 times the grain size (*d*_*g*_ ≈ 25–35μm for the examined SS alloy). Furthermore, Figure 2 shows also how a well‐cast surface replica exhibits similar characteristics to the original surface. On the other hand, a bad surface replica with entrapped air bubbles deviates from the original surface characteristics and exhibits different correlations. This phenomenon is very similar in appearance to dots formed during the casting of tool marks in human cartilage, where a very different underlying mechanism arise from cartilage cavities, which are areas where the strong collagen fibers leave space for the chondrocytes [19].

To highlight these characteristic length scales and their impact on the forensic comparison of the fracture surface, Figure 1C illustrates the role of the proper comparison band of the wavelengths (unique character) within the fracture surface topology. The three lines represent the marked line A–A at the center of the pair of processed original Base and Tip surface images and the Tip’s replicas (see Figure 1B). The pair of images was processed by the mathematical Fast Fourier Transform (FFT) operator. In one set, the low‐frequency content (large wavelength, *λ* > 100 μm) was retained, as highlighted in the middle plot of Figure 1C. The three profiles showed a perfect match and asserted the uniqueness of the topology in this range. In the other set, the high‐frequency content was retained (small wavelength, 2 μm ≤ *λ* < 20 μm), as highlighted on the lower plot of Figure 1C. The line comparison was quite random with no similarity, indicating the self‐affine deformation at this range. This trend is similar to that of an optical image obtained by high magnification and a small field of view, where the local fracture mechanism shows similar topological surface features over the fractured surface with indistinguishable character. Accordingly, the utilization of the observation length scale, λ for the unique fractured surface texture provides the proper level of details with the proper microscope magnification.

In this paper, a set of fracture pairs and their replica were examined via 3D imaging profilometry and then analyzed with statistical decision‐making tools. We quantify the range of features’ resolution that generic silicon‐type casting materials can capture to replicate the fracture topology. In particular, we identify the ranges of wavelength and frequencies that it replicates to perform quantitative forensic fracture matches. In optical microscopy, it will set the proper magnification to view the replicated surface and the number of features or events that can be identified for matching purposes. For this purpose, we acquired six overlapping topological images of each fracture surface (Base and Tip) along with the Tip’s replicas and obtained correlations for each of the three comparison pairs along with two frequency ranges and six overlapping images to obtain a matrix‐valued feature that can be used to distinguish between matching and non‐matching pairs of images. To classify these matrix‐valued features, a quadratic discriminant analysis (QDA) classification algorithm with the matrix *t*‐distribution is trained on a separate set of samples of the same material‐class and imaged by the same operator. This classifier is then used to classify the three surface pairs: (i) the original Base with Tip, (ii) Tip’s casted replicas with original Base, and (iii) Tip’s casted replicas with original Tip, resulting in perfect classification in all cases. The details of materials and methods are presented in Section 2, highlighting sample preparation and surface replication, fracture surface imaging protocol, surface spectral analysis, and a summary of the statistical model. The results and findings are summarized in Section 3 and followed by concluding remarks in Section 4.

## 2 MATERIALS AND METHODS

### 2.1 Fracture samples and replica generation

**FIGURE 3:**
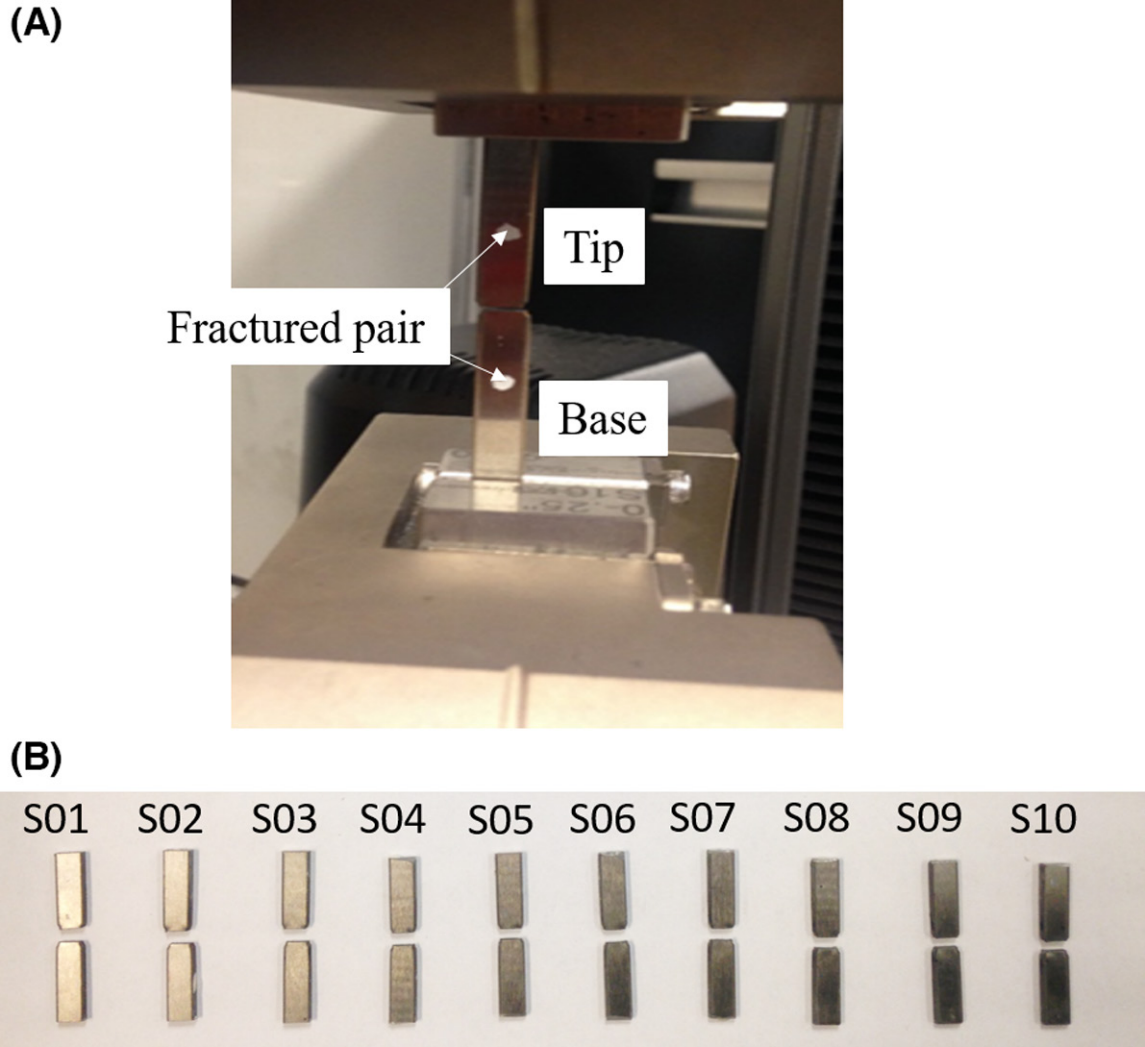
(A) Sample set of steel rods fractured under controlled tensile loading, (B) a set of 10 fracture pairs, providing the Base and Tip sections of the fractured steel rods utilized in this study

A set of 10 rectangular (0.25″ wide and 1/16″ thick) rods of a common tool steel material (SS‐440C) and cut from the same metal sheet to minimize any variability from the manufacturer was used in this study. The steel rods were loaded in an INSTRON 8862 servo‐electric computer‐controlled testing frame under a controlled extension of 1 mm/min displacement rate till fracture. The fractured pair surfaces were coded as a “Base” and a “Tip,” as shown within the loading grips of Figure 3A. The set of 10 fracture pairs are shown in Figure 3B.

**FIGURE 4:**
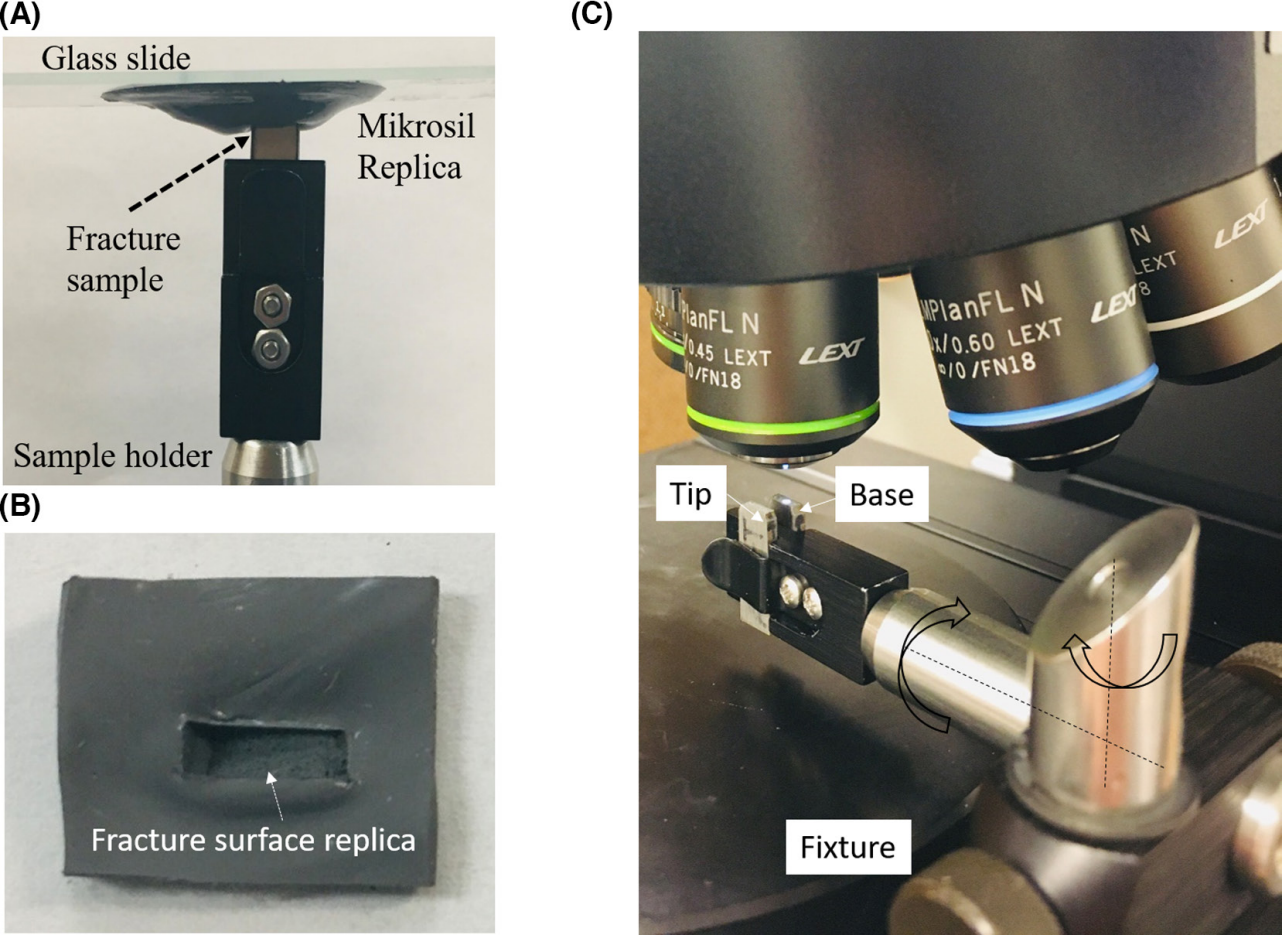
Replication process and imaging of the fracture surface. (A) Vertical replication process of the fracture surface utilizing a gray silicone type casting material, (B) glass slide containing the replica of the fracture surface, (C) fracture surface characterization using the laser confocal microscope showing Base and its Tip alignment

A Mikrosil gray silicone type casting material was utilized in generating the fracture surface replicas because it is one of the common replication materials for forensic analysis [19, 42, 45]. The Tip surface was replicated for the entire set. One of the major troublesome issues was the appearance of air bubbles within the image field of view. The air bubbles ranged in size from 70–200 μm, which greatly interfered with the analysis. Figure 2 shows the effect that a bad replica has on biasing the domain of unique surface textures to be used for the comparative analysis. The replica surface with bubbles failed to show unique textures within the FOV and thereby could not be used for the comparative analysis. A glass slide with a uniform layer made of a silicon‐based replica material was applied upside down, to the fracture surface, as in Figure 4A,B, to replicate the fracture surface. The glass slide was gently eased onto the surface with a 45° inclination to eliminate or minimize the entrapment of air pockets. Acetone droplets were applied to the resin and the hardener mixture at the ratio in 1:10 by volume to reduce the propensity for bubble entrapment during the replica process. The dilution process reduced the viscosity of replica paste and greatly improved its flowability and enabled capturing the fine details of the fracture surface, while providing a bubble‐free replica surface, as shown in the set of images of Figure 2A. The replica cast was left on the sample surface for about 15 min to be fully hardened. The images in Figure 1A,B show a Base‐Tip fracture pair and the replica of the Tip, which exactly matches both the Base and the Tip. The Tip for each of the 10 fracture pairs was replicated and utilized to form three groups for comparative analysis, namely (i) Base‐Tip, (ii) Base‐Replicas, and (iii) Tip‐Replicas.

### 2.2 Fracture surface imaging

All 3D fractured surface height topological maps were acquired by a 3D confocal laser microscope (OLYMPUS LEXT‐OLS5000). The microscope provides optical magnification of 5–100× with additional digital multiplication of 9×. For the purpose of classification of matching and non‐matching surfaces, the observation scales (imaging window) should be controlled by the self‐affine transition length scale, which should cover at least 10 periods of the fracture process zone or about 20–30 grain diameters to avert signal aliasing. The transition length scale is shown in Figure 2B, beyond which a saturation in the height‐height correlation is seen and indicative of unique surface texture. Accordingly, we utilized 20× objective, which maps the measuring array of 1024 × 1024 pixels to a 640 μm FOV with 0.625 μm/pixel spatial inter‐point resolution.

The topological imaging protocol includes an alignment step and an image‐adjustment step. The alignment step ensures that the pair of fractured surfaces to be analyzed are aligned relative to each other without planar misalignment (in‐plane tilt of the pair of surfaces), which could significantly deteriorate the correlation of the pairs of surface spectra. In optical comparative microscopy, the pair of fractured surfaces to be compared are viewed simultaneously and tilt adjustment done visually. For 3D topological comparison, such alignment step can be done mathematically [57], adding an extra step of complexity. To streamline the imaging process, a fixture was developed to hold the pair of fracture surfaces parallel and aligned to each other, as shown in Figure 4C. The fixture also allows rotational movements around two axes to accommodate non‐planar titled fracture surfaces. The image‐adjustment‐step entails adjusting the imaging volume (in direction normal to the fracture surface) to capture the entire topological surface height range of the fractured surface within the field of view. The laser intensity was then adjusted to map the entire surface topology within the dynamic range of the optical sensor without imposing over‐saturation and truncation of extreme (high tortuosity) topological details. A standard mathematical out‐of‐plane tilt is applied to all images to remove global fracture surface tilt. Furthermore, standard mathematical spike noise removal is also applied to remove any measurements that is beyond one standard deviation from the average of the surrounding window of 7‐pixel radius. The noisy pixel is replaced with the surrounding window average. It should be noted that the range of wavelength of interests (essential topological features for comparison) are mapped to about 50–100 pixels, rendering the correction window to be less than one quarter of the smallest recorded wavelength, and thereby not affecting any topological feature of interest.

**FIGURE 5:**
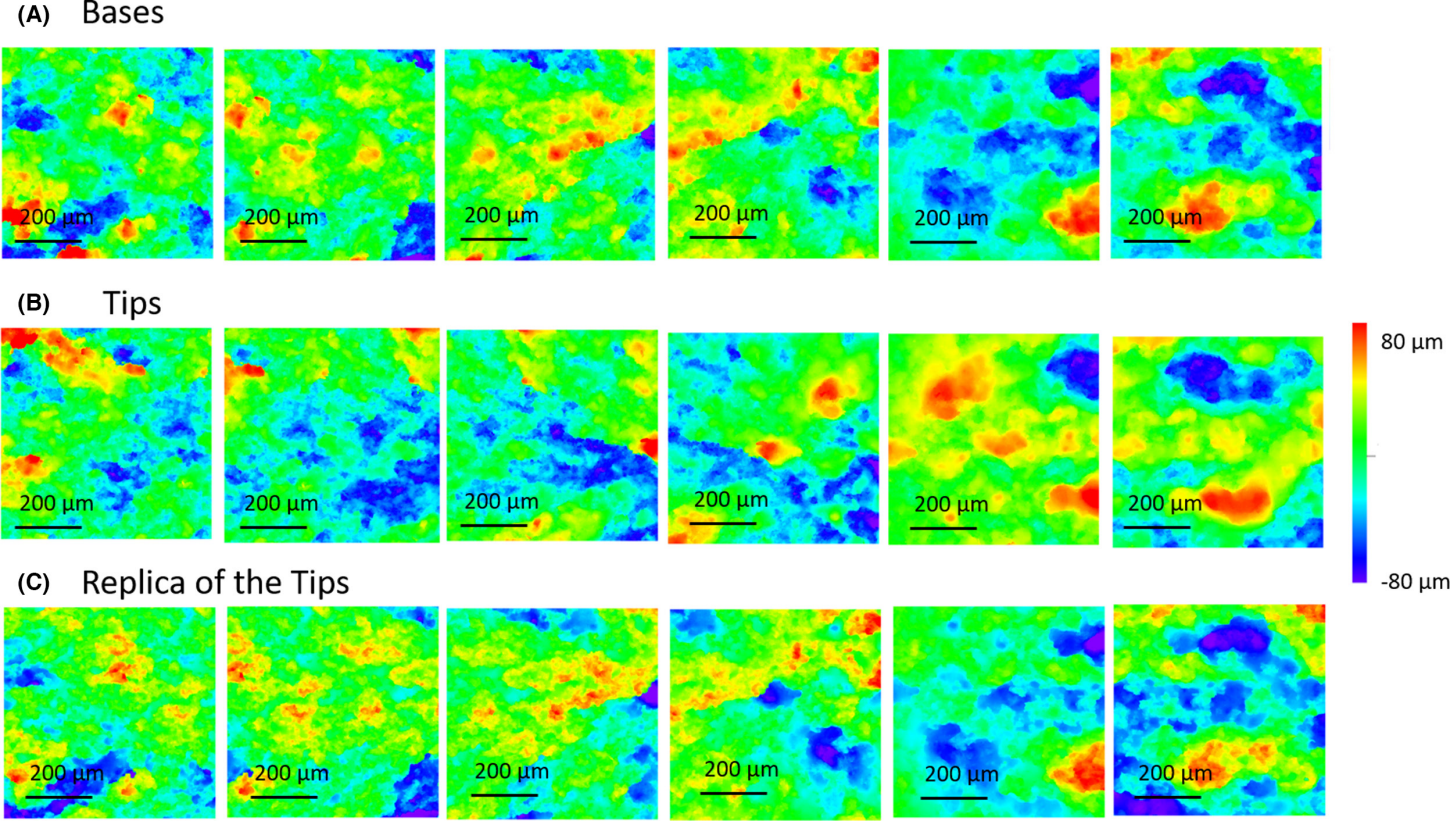
A representative topological set of images for a fracture pair for the acquired 6‐images at 50% overlap and 20× objective. (A) Base, (B) Tip, and (C) a replica of the Tip

All images were acquired relative to a reference mark, which is the corner of the sample. The first image reference coordinate, marked by the white box on Figure 1A, starts at a 1000 μm from the right vertical edge and is approximately centered around the centerline of the sample thickness. For each fracture pair, a set of six successive images with 50% overlap was acquired for both the Tip and the Base. Our previous analysis [47] showed that a set of six images with 50% overlap is required to get classification with very high probability, and to ensure diminishing probability of misclassifications. Since the FOV was controlled by the saturation scale of the height‐height correlation, multiple images were needed to overcome the imaging noise generated by missing grains between the pair of the fracture surface and the special circumstances of complex tortuous path of fracture. It should also be noted that having a super‐image of stitched smaller images with the selected FOV, equivalent to the five individual images, results in misregistry at the overlapping boundary of the stitched images. The ambiguity within the overlapping region will lead to an additional interfering frequency within the band of comparison. Additionally, it is more computationally practical to perform FFT operation on smaller arrays as the operation scales with *2* ^*n*^, where *n* is the image size. Figure 5 shows a sequence of six topological images for a Base‐Tip fracture pair and the replica of the Tip.

### 2.3 Spectral analysis and image correlation

The FFT operator was applied to each image to generate the corresponding frequency‐space representation and the image topological spectral contents. A mathematical Hann filter with 10% edge smoothing ratio was applied to the original image to provide a periodic boundary for the image edges, before the FFT operator. The frequency‐space analysis provides the ability to segment the surface topological frequency ranges for comparison. For instance, the lower frequency bands represent the macro‐fracture features and the unique river marks. The high‐frequency bands represent the micro mechanism of the fracture process as depicted in the 2‐D topological height profiles shown in Figure 1C for line A–A showing the original topological details, and the decomposition of the long and short‐wavelength components. For statistical comparison and decision‐making, the statistical correlations between the spectra of each of the pair of surfaces are computed within banded frequencies, with increments in the bands determined by the scale of the image and the microstructure of the material, yielding a similarity measure on each frequency band for the corresponding pairs of images.

**FIGURE 6:**
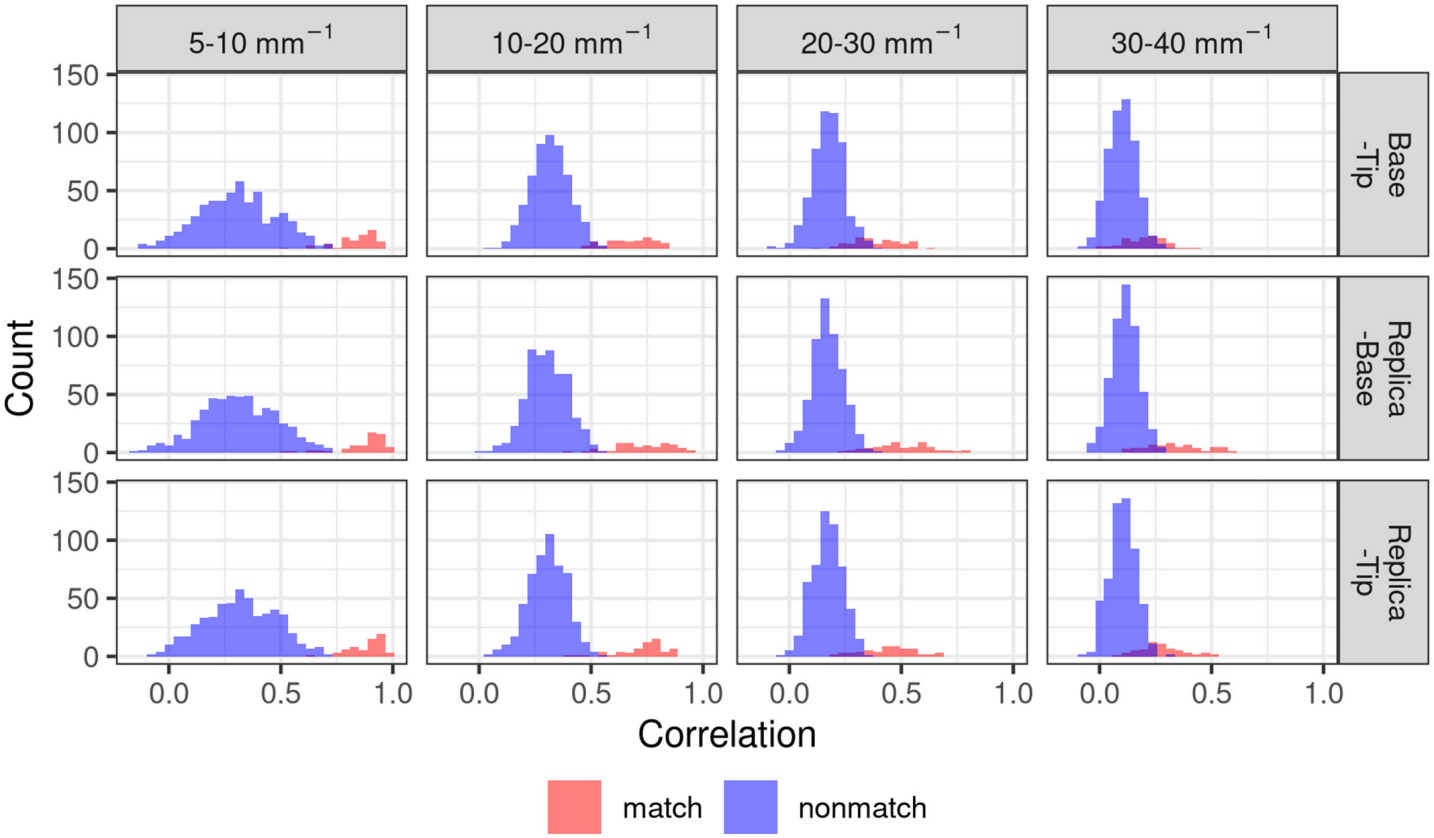
Correlation histograms for true matches and true non‐matches for different frequency bands and surface‐pair comparison. Lower frequencies are well separated, while higher frequencies start to have overlap

**FIGURE 7:**
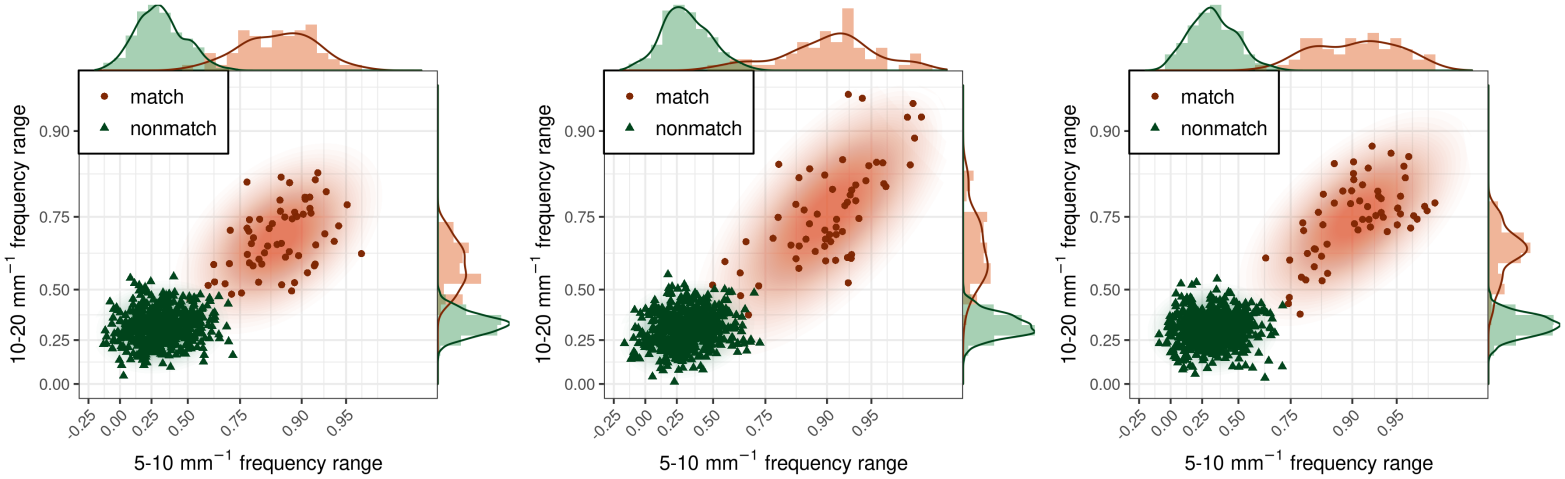
Scatterplots of fracture surface correlations in the Fisher‐Z transform scale, and for the three sets of surface‐pair comparison. Combining frequency bands increase the overlap between matches and non‐matches. In each plot there a total of 60 matched pairs and 540 unmatched pairs. Furthermore, Fisher‐*Z* correlations form ellipsoids for both matches and non‐matches, and therefore, make them suitable for a multivariate elliptical model such as the multivariate normal or *t*

Comparison of image pairs for when the Tip and Base surfaces were from the same rod are called true matches, while comparison of surface pairs from different rods are called true non‐matches. Figure 6 shows the correlation distribution for comparison made on pairs of images from matching and non‐matching fracture pairs to estimate the distribution for both true matches and true non‐matches. The image data set was derived from 10 Base and Tip pairs and six images from each surface, resulting in 100 pairs of Base‐Tip combinations $\left(\begin{array}{c} 10\\ 2 \end{array}\right)$. Note that, matching the Base of sample A with the Tip of sample B is not identical to matching the Base of sample B with the Tip of sample A (since the Tip and Base of a fracture surface are not perfect mirror images of each other due fracture surface irregularities between a Tip‐Base pair). Accordingly, there are 60 matched image pairs (10 matched pairs × 6 sets of image pairs per fracture pair) and 540 non‐matched image‐pair comparisons (90 non‐matched pairs × 6 sets of image pairs per fracture pair). Correlation analysis shows a clear separation between the true matches (red color in Figure 6) and true non‐matches (blue color in Figure 6) for the 5–10 and 10–20 mm^−1^ frequency bands. The distributions start to be less discriminating and overlap more at higher frequencies. The overlap between matches and non‐matches can be further reduced by combining the two most discriminating bands of 5–10 and 10–20 mm^−1^, as seen in the displays of their correlations in the Fisher‐Z transform (the inverse hyperbolic tangent) scale (Figure 7). According to the contours, the surface pair with the least overlapping matches and non‐matches is replica‐Tip, which highlights the effectiveness of the surface replicas in capturing the important relevant information for classification.

### 2.4 Model training and classification

The correlations that result from comparing each surface pair can be arranged in a 2×6 matrix, where the two rows correspond to the two relevant frequency bands of 5–10 and 10–20 mm^−1^ and the six columns to the six overlapping images taken on the surfaces. To classify a 2×6 matrix X as a match or non‐match, we first apply the Fisher‐Z transform to each element of X and then use Bayes' theorem to obtain the posterior probability that X is a match as

(1) $$P\left(X=\text{match}\right)=\frac{p_{1}\;f_{1}\left(X\right)}{p_{1}\;f_{1}\left(X\right)+\left(1-p_{1}\right)f_{2}\left(X\right)},$$

where *f*_1_ and *f*_2_ are the probability density functions (pdfs) of the true matches and non‐matches, respectively, and *p*_1_ is the prior probability that X is a match. We set *p*_1_ = 0.5 because then the log‐odd of the posterior probability in Equation (1) simply becomes the log of the likelihood ratio, which is easily interpretable. The posterior probability Equation (1) involves the unknown functions *f*_1_ and *f*_2_, with parameters that we estimate using a training sample of 10 rods of the same material and fractured the same way as the studied steel rods, and that were imaged by the same operator who imaged the steel rods surfaces and their replicas. While we followed all these restrictions in generating the training set; however, we found that any set of fractured samples with the same range of grain sizes and similar brittle fracture mechanism results in the same quality of classifications [47]. To estimate *f*_1_ and *f*_2_ we fitted the matrix‐variate *t*‐distribution [58] with 5 degrees of freedom to both the true matches and true non‐matches in our training sample using the expectation–maximization algorithm [46], which generalizes the block‐relaxation algorithm given in [59]. Our matrix‐variate‐*t* model postulates a mean matrix with identical columns and a correlation structure across the six overlapping images that is dictated according to an AR(1) autoregressive process [47].

After estimating the parameters of the pdfs for *f*_1_ and *f*_2_, we proceed to estimate the posterior probability that a 2×6 matrix X corresponds to a matching pair of surfaces as per Equation (1). In this study, the classification decision was made at the *p* = 0.5 level, meaning that posterior probabilities greater than 0.5 correspond to matching surfaces. The log‐odds ratio of the posterior probability in Equation (1) can also be converted to a log‐likelihood ratio, which can be used as a measure for forensic evidence comparison [2, 8, 25, 44, 45, 60].

### 2.5 Assessment of replica effectiveness in wavelength recovery and topological mapping

In the previous section, we correctly classified every image pair using a QDA classification algorithm based on the matrix *t*‐distribution with 5 degrees of freedom. For the purpose of studying the relevant wavelengths recovered from our replicas, we used the matrix *t*‐distribution to obtain the mean correlation along with the 10 frequency bands defined by the thresholds of 3‐5‐10‐20‐25‐33‐50‐67‐100‐133‐200 mm^−1^. This corresponds to wavelength bands of 333‐200‐100‐50‐30‐20‐15‐10‐7.5‐5 μm, respectively. For the Tip‐Replica pairs, we obtained the correlations from the six overlapping images for all the specified frequency bands to obtain 10 matching pairs and 90 non‐matching pairs of surfaces. Then, we formulated 10×6 matrix features, with the 10 rows corresponding to the frequency bands and the 6 columns to the overlapping images. We fit the matrix *t*‐distribution with 5 degrees of freedom, AR(1) correlation structure along with the columns to capture the overlapping context of the images, and identical columns across the mean matrix. We note that the underlying fracture process generating the topography was not spatially stationary, meaning that at different positions within the fracture surface, the frequency of the fracture process varied slightly due to the inherent randomness of the microstructure. The FFT operator integrates over the entire 2D space, but because of non‐stationarity, there are slight phase differences at different locations of the fracture surface. These local phase differences give rise to destructive interference between frequencies that really ought to be correlated, especially at the high‐frequency range. This phenomenon is an inherent limitation of the FFT operator. To study the effect of the noise in the Fourier space on the mean correlations, we applied a 3‐point kernel filter to the FFT spectra of each topological image before obtaining the correlation coefficients. The kernel used was a 3 × 3 spatial‐frequency‐sample blur. Then, we fit the matrix *t*‐distribution. Note that the blur kernel has an effect only on the high frequencies; therefore, it does not affect any of the match analysis utilizing the low‐frequency components as explained in Section 2.4.

## 3 RESULTS AND DISCUSSION

**FIGURE 8:**
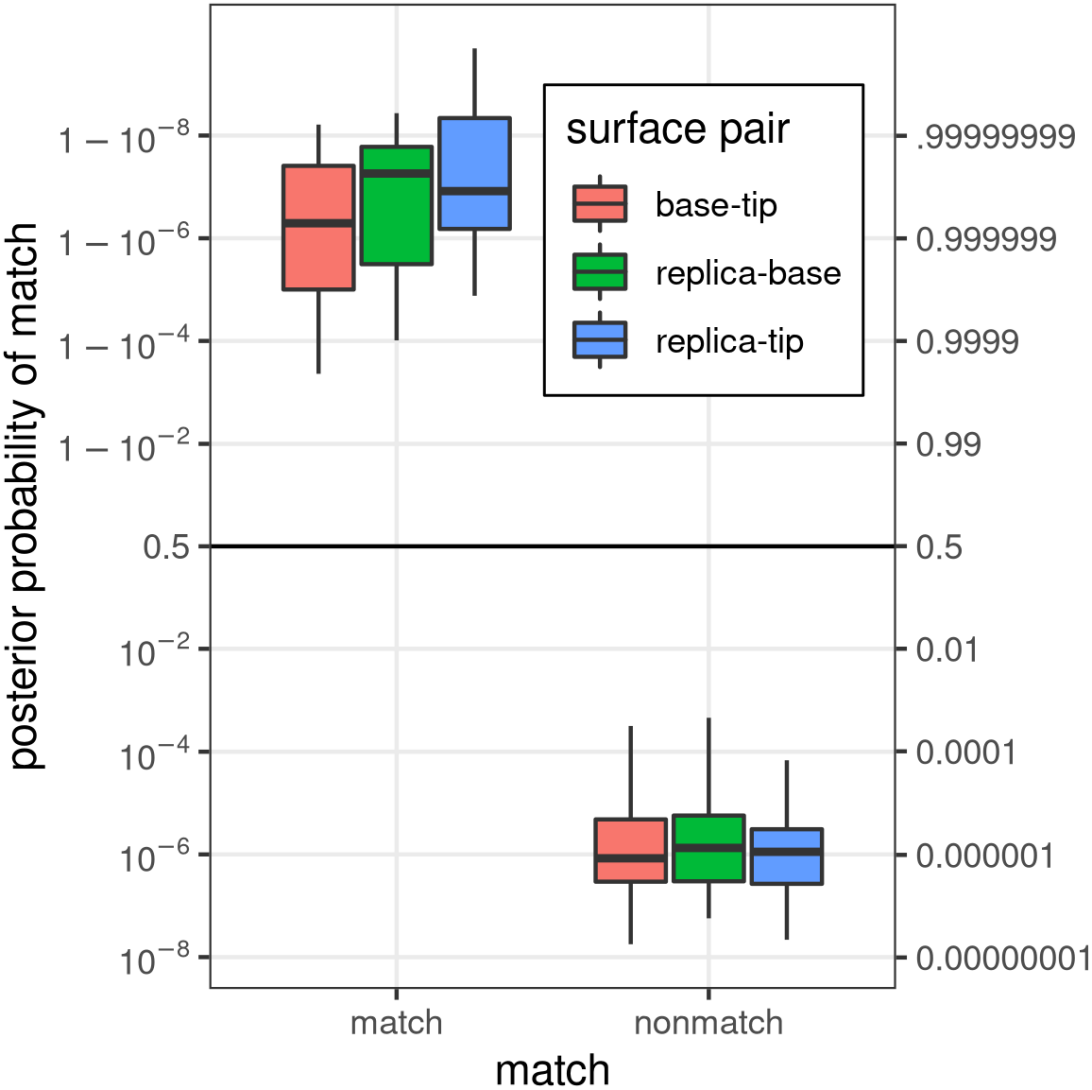
Posterior probability of being a match (in log‐odds scale) using a model trained on a separate set of images from the same surfaces and tested on the pair of surfaces of Base‐Tip, replica‐Base, and replica‐Tip. Higher values indicate stronger evidence of a match

Our framework utilizes a statistical model to produce a likelihood ratio or log‐odds ratio of a matching pair or set of pairs; in addition, our model can estimate the probabilities of misclassification. Other probabilistic models such as the likelihood ratio and Congruent Matching Cells are used in many forensic applications such as fingerprint identifications and bullet matching [35, 57, 61, 62]. Our focus also is on examining the potential of replicas to transfer all the topological details required for the analysis at the proper magnifications. The classification model was applied to three sets of pairs of surfaces: the original Bases with Tips, the Bases with the Tip replicas, and the Tips with their replicas. Figure 8 shows the posterior probabilities of match obtained on the three sets of surface pairs in the log‐odds scale. Larger posterior log‐odds indicate more evidence that a surface pair is a match, whereas lower log‐odds indicate more evidence that a surface pair is a non‐match. Utilizing the *t*‐distribution with 5 degrees of freedom provides great confidence in the discrimination power of the proposed comparison and statistical analysis framework. The model classified the three cases with 10 pairs of true matches and the 90 pairs of true non‐matches with no false negatives and no false positives. That is, there are a total of 30 pairs of true matches and 270 pairs of true non‐matches. The 90 replicas show a high probability of match when compared to the original fractured surfaces. This high accuracy exists for both original Base‐Tips, replicas‐Bases, replicas‐Tips, although the replicas were cast only on the surfaces of the Tips. These results demonstrate the ability of the replicas to capture the relevant features that are important for discrimination. Furthermore, for the true match group, the lowest posterior probability was higher than 0.9996, while the highest posterior probability for the true non‐match was less than 0.005. The stark difference between the match and non‐match probabilities highlights the strength of using the physical basis of fracture mechanics to guide the imaging procedure and construct the statistical discrimination framework.

**FIGURE 9:**
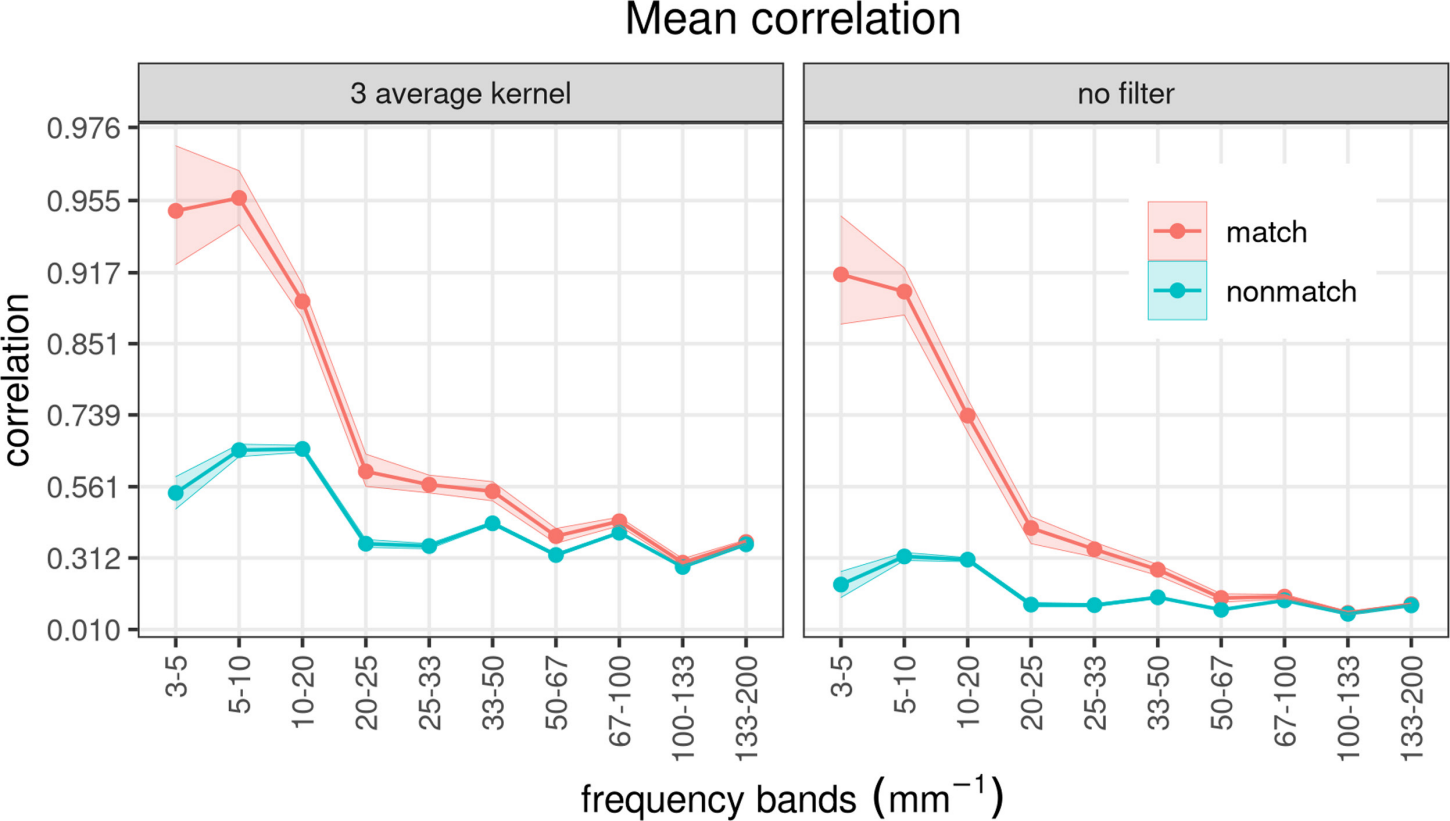
Mean correlations obtained from the comparison between the tip and its replica along with 10 frequency bands and to both matches and non‐matches, and for both the original and filtered frequency spectra. The matching correlations decrease with increasing frequency bands while still being distinctively different from the non‐matches until it reaches frequency bands less than 100  mm^−1^ (short wavelength greater than 10 μm)

Figure 9 summarizes the results for the replication capacity of the silicone replica technique for all the frequency bands in the range of 3–200 mm^−1^ or the corresponding wavelength of 333–5 μm, respectively. The mean correlations are shown for each of the comparison bands along with 95% bootstrap confidence intervals for the group of match and non‐match cases. The results are shown for both the original and the filtered frequency spectra of fractured sample Tips and their casted replicas. Figure 9 shows that with increasing frequency bands (i.e., reduction of the wavelength in real space), the match correlations between the Tip and its replica decay in magnitude and spread, indicating loss of differentiation of unique events. Furthermore, the correlations obtained from the filtered FFTs have increased correlations in both the matches and the non‐matches. This is equivalent to using a discrimination framework at higher magnification where the fracture surface topology is self‐affine and indistinguishable from one surface to another of the same class. However, Figure 9A shows that the match correlation remains around 0.4–0.5 for a high frequency of 100 mm^−1^ or a short wavelength of 10 μm. This result gives confidence that replicas can still reliably reproduce wavelengths down to the micron ranges. However, such a range needs additional investigation with a smaller FOV and large magnification, employing a master surface with well‐defined micron range features. Also, at large wavelengths, that is, at wavelengths greater than about a fifth of the imaging window size, Figure 9A shows a slight drop in correlation at the 3‐5 mm^−1^ frequency band when compared to the 5–10 mm^−1^ frequency band. This is a limitation of the discretization process wherein the resolution per frequency line is 1.56 mm^−1^, which will provide a very limited number of data points (about 7 discretized frequency lines) in the 3–5 mm^−1^ band. A larger FOV at the same magnification would be required to refine the frequency‐band lines and resolve these long‐wavelength limitations.

Our analysis provides two significant results. *First*, the analysis supports our previously developed classification procedure [47]. It shows how replicas effectively capture surface fractures along with wavelength topological details in the range of the two frequency‐band analyses of 5–10 and 10–20 mm^‐1,^ equivalently between 200–100 and 100–50 μm wavelengths. The topological features at these length scales are unique and helpful for distinguishing between matches and non‐matches. *Second*, the analysis shows the ability of the replica to faithfully replicate fracture features with wavelengths all the way to the 20 μm range. For forensic comparison, the replicas are well suited for mapping features of 20 μm and larger. It can be assertively stated that the replicas effectively distinguish matches from non‐matches in low‐frequency ranges, and that they stop being distinctively different for frequencies above 100 mm^−1^, where the micro‐features of the local fracture processes that are common to both matches and non‐matches are compared.

While our presented data set was acquired on a well‐controlled fracture event of stainless steel rods, the results are general and applicable to crime scene scenarios. Here, we have attempted to eliminate other interfering issues that might bias the conclusion in gaging the ability of casted silicon replica to capture surface features to the micron‐scale. However, we have examined a wide range of metallic articles with a range of microstructure grain sizes of 10–30 μm, covering a broad range of engineering metallic alloys. We found that regardless of the origin and nature of the training set, we were able to acquire very high posterior match probabilities well above 99.9+% for true match pairs. For non‐match pairs, we consistently achieved very low posterior match probabilities well below 1%. Our framework is also applicable to different classes of materials including ceramics and automotive plastic components. Our preliminary work showed similar levels of discrimination, though the imaging and comparison scales were adjusted to the relevant fracture process zone size. While this reassures the confidence in the prospects of applying our framework, additional investigations are needed for each of these classes of materials.

While our statistical analysis framework is quite promising for a wide class of material comparison and examination, the limitations of our technique arise from the ability to produce accurate 3D topological representations of the fracture surface for comparison. There are two main limitations for the technique.

(1) The framework presented here is accurate and feasible for the class of materials that exhibit brittle or semi‐brittle fracture, wherein the fracture surface is relatively planar within several hundred microns. The limitation here is having the imaging depth resolution in the sub‐micron range for the entire surface topology range. If the fracture surface exhibits large tortuous‐paths with topological variation in the millimeter‐range, additional mathematical treatments would be required, similar to comparison of cylindrical surfaces (e.g., cartridge cases) [36, 63].

(2) If the fracture surface has many missing grains or groups of grains, these missing topological details will greatly affect the fracture‐pair correlations and the decision‐making process. In the current work, we found that the use of six images with 50% overlap would tolerate the loss of several grains within the FOV, with a total area density of about 10%. If the differences between the pairs of images are larger than this range due to grain fall‐out or excessive corrosion, larger image sets will be required. This is a problem of focused investigation and will be reported after analysis of different relevant scenarios.

Within the limitations noted above, the developed framework establishes the basis of forensic comparison of fractured articles and their replicas while providing a reliable quantitative statistical forensic comparison. The framework utilizes the foundation of fracture mechanics to establish the FOV and scales of comparisons of the fracture surface topology of fragment pairs.

## 4 CONCLUSIONS

We have utilized our developed quantitative statistical comparative analysis framework to examine the potential of the cast replicas in the comparative forensic analysis of topological details of pairs of fractured surfaces. The replica surfaces faithfully reproduce the topological details with wavelength features greater than 20 μm. The replicas showed a high probability of match when compared to the original Base‐Tip fractured pairs. This result highlights the replicas' ability to capture the relevant features that are important for discrimination. Furthermore, the stark difference between the match and the non‐match probabilities highlights the strength of using the physical basis of fracture mechanics to guide the imaging procedure and construct the statistical discrimination framework. The presented framework has a high potential in assisting forensic scientists in providing conclusive decision‐making with quantifiable probabilities for a wide range of fractured and broken forensic articles along with their replicas. All of the classification scores were higher than 99.96%, and were the highest for the Tip‐replica comparison pair, demonstrating the potential for using replicas, relative to the original Base‐Tip comparison. The underlying correlations, which are strong for the low‐frequency bands capturing the macro‐fracture features, indicate the potential of using replicas to reproduce the relevant features present in forensic fracture evidence.

## Supporting information


**Appendix** **S1**
