# Supplementary material for: Quantitative matching of forensic evidence fragments utilizing 3D microscopy analysis of fracture surface replicas
Source: J Forensic Sci. 2022 Mar 7;67(3):899–910. doi: 10.1111/1556-4029.15012 (PMC9311802; doi:10.1111/1556-4029.15012)
Supplement: Supplementary file 1 — Appendix S1 [file JFO-67-899-s001.pdf]

# Code for Statistical Analysis of "Quantitative Matching of Forensic Evidence Fragments Utilizing 3D Microscopy Analysis of Fracture Surface Replicas"

Carlos Llosa-Vite

This document presents all the statistical code and code output that was used in the paper submitted to the Journal of Forensic Sciences titled "Quantitative Matching of Forensic Evidence Fragments Utilizing 3D Microscopy Analysis of Fracture Surface Replicas". This includes code for generating correlations from raw FFTs, classifying match/nonmatch based on the correlations, generating figures, and applying a filter to the FFTs. The code requires our R package `topologymatcheR`, which has routines for generating correlations, classification, and some graphs. The R package is available on `github.com/carlos-llosa/topologymatcheR`.

Section 1 describes the correlation files that were used for the statistical analysis of Section 2, and that were generated from the code of Section 3. Section 2 presents the code that performs the statistical analysis involved in Figures 6-9. Section 3 presents the code that was used for generating correlation features from raw topological FFTs, and Section 4 applies a 3-cross averaging filter to the raw FFT data. The code for Sections 3-4 was run on the terminal, and here we present the .Rout files.

## Contents


**1 Data** **2**

**2 Code for generating figures** **3**
- 2.1 Figure 6 . . . 3
- 2.2 Figure 7 . . . 6
- 2.3 Figure 8 . . . 12
- 2.4 Figure 9 . . . 16

**3 Code for generating correlations** **20**
- 3.1 For base-tip, replica-base and replica-tip (for Figures 6-9) . . . 21
- 3.2 For averaged replica-tip (for Figure 9) . . . 28

**4 3-cross averaging of raw FFT data** **31**

# 1 Data

The code for generating Figures and performing statistical analysis depend only on correlation csv files, which are available at `github.com/carlos-llosa/topologymatcheR/tree/main/data`. The five csv files are described here in detail:

**base-tip-corrs.csv:** This files contains correlations along 12 frequency bands, 10 fracture surfaces and 6 overlapping images. The correlations compare the base with the tip. This csv file is used in Figures 6-8. This correlation file was generated from raw topological FFTs using the code in Section 3.1.

**A3-corrs.csv:** This file is similar to `base-tip-corrs.csv`, except that the images that were used to generate these correlations were obtained on a separate session by the same microscope operator. We use these correlations as the training for the classification algorithm behind Figure 8.

**replica-tip-corrs.csv:** This files contains correlations along 12 frequency bands, 10 fracture surfaces and 6 overlapping images. The correlations compare the tip with the replica of the tip. This csv file is used in Figures 6-9. This correlation file was generated from raw topological FFTs using the code in Section 3.1.

**3crossavg-replica-tip-corrs.csv:** This file is similar to `replica-tip-corrs.csv`, except that the raw FFTs that were used to generate these correlations were applied an averaging window filter to reduce noise. This file is used in Figure 9, where the effect of the noise (and the filter) is studied in detail.

**replica-base-corrs.csv:** This files contains correlations along 12 frequency bands, 10 fracture surfaces and 6 overlapping images. The correlations compare the base with the replica of the tip. This csv file is used in figures 6-8. This correlation file was generated from raw topological FFTs using the code in Section 3.1.

# 2 Code for generating figures

## 2.1 Figure 6

Here we present the code that generates Figure 6. Figure 6 presents histograms for the correlations along 4 frequency bands and for the three comparison pairs of base-tip, replica-base and replica-tip. The correlations are generated in Section 3.1, and the Figures are generated using the R package ggplot2.

# Figure 6

```r
library(ggplot2)
loc <- "~/Desktop/forensics/carlos-reports/writes/replica/codeNdata/generating-correlations/"
base_tip <- read.csv(paste0(loc,"base-tip-corrs.csv"))[,c(3:6,16)]
rep_base <- read.csv(paste0(loc,"replica-base-corrs.csv"))[,c(3:6,16)]
rep_tip <- read.csv(paste0(loc,"replica-tip-corrs.csv"))[,c(3:6,16)]

names(base_tip) <- names(rep_base) <- names(rep_tip) <- c("f5_10","f10_20","f20_30",
                                                          "f30_40","match")

base_tip <- reshape2::melt(base_tip)
```

```
## Using match as id variables
```

```r
rep_base <- reshape2::melt(rep_base)
```

```
## Using match as id variables
```

```r
rep_tip <- reshape2::melt(rep_tip)
```

```
## Using match as id variables
```

```r
base_tip$replica <- "base\n-tip"
rep_base$replica <- "replica\n-base"
rep_tip$replica <- "replica\n-tip"

alldat <- rbind(base_tip,rep_base,rep_tip)
my_labeller <- as_labeller(c(f5_10="'5-10 mm'^-1",f10_20="'10-20 mm'^-1",
                             f20_30="'20-30 mm'^-1",f30_40="'30-40 mm'^-1"),
                           default = label_parsed)


p1 <- ggplot(alldat, aes(x = value, fill = match)) +
      geom_histogram(alpha = 0.5, position = "identity", bins = 30) +
      facet_grid(replica~variable, labeller = labeller(variable = my_labeller))+
      theme_bw()+
      xlab("Correlation")+
      ylab("Count")+
      labs(fill = "Match")+
      scale_fill_manual(values = c("#FF0000","#0000FF"))+
      scale_x_continuous(breaks = round(seq(0,1,length.out = 3),2))+
      theme(legend.position="bottom",
            legend.box.margin=margin(-10,-10,-10,-10))
p1
```

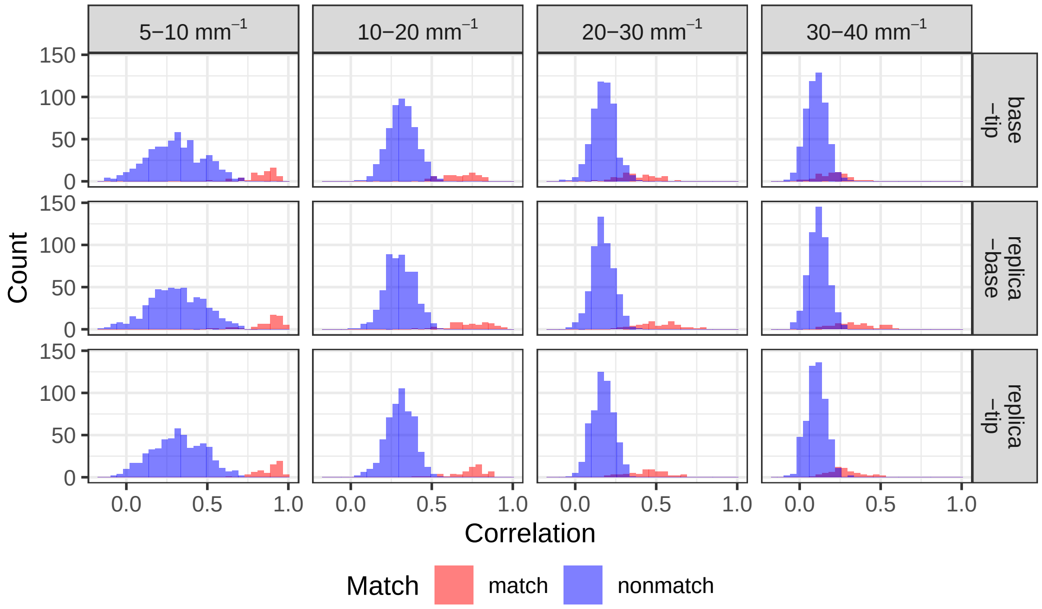


## save

```
ggsave("/home/carlos/Desktop/forensics/carlos-reports/writes/replica/codeNdata/figures/fig6/fig6.tiff",
       p1,   # The directory you want to save the file in
       width = 6, # The width of the plot in inches
       height =3.5)
```

## 2.2 Figure 7

Here we present the code that generates Figure 7. Figure 7 presents a scatter plots in the Fisher-z scale of the correlations, with 5-10 frequency in the x-axis and 10-20 frequency in the y-axis. This plot also includes histograms along the y-axis and x-axis. The plots here were generated using the R package ggplot2, and arranged using the R package ggpubr.

# Figure 7

```
library(ggplot2)
library(dplyr)
```

```
##
## Attaching package: 'dplyr'

## The following objects are masked from 'package:stats':
##
##     filter, lag

## The following objects are masked from 'package:base':
##
##     intersect, setdiff, setequal, union
```

```
library(magrittr)
library(ggpubr)
library(cowplot)
```

```
##
## Attaching package: 'cowplot'

## The following object is masked from 'package:ggpubr':
##
##     get_legend
```

```
library(scales)
library(ellipse)
```

```
##
## Attaching package: 'ellipse'

## The following object is masked from 'package:graphics':
##
##     pairs
```

```
fisher_trans = function() trans_new("fisher", function(x) psych::fisherz(x),
                                    function(x) psych::fisherz2r(x))
pair <- "base-tip"

plot_gen <- function(pair){

  loc <- "~/Desktop/forensics/carlos-reports/writes/replica/codeNdata/generating-correlations/"
  #pair <- "base_tip"
  #pair <- "rep_base"
  xlimm <- c(-.2,.985)
  ylimm <- c(0,.94)
  totalcorset = read.csv(file =   paste0(loc,pair,"-corrs.csv"), check.names = FALSE,
                         header = TRUE)[,-1]

  totalcorset$x = totalcorset$`5-10`
```

```r
totalcorset$y = totalcorset$`10-20`


########## variances (in transformed space)

totalcorset$zx = atanh(totalcorset$x )
totalcorset$zy = atanh(totalcorset$y )

matchz = totalcorset %>% filter(match == "match") %>% select(zx, zy) %>% colMeans
nonmatchz = totalcorset %>% filter(match == "nonmatch") %>% select(zx, zy) %>% colMeans

matchzdf = data.frame(zx = rep(matchz[1], 100), zy = rep(matchz[2], 100))
nonmatchzdf = data.frame(zx = rep(nonmatchz[1], 100), zy = rep(nonmatchz[2], 100))


matchmean = tanh(matchz)
names(matchmean) = c("x", "y")

nonmatchmean = tanh(nonmatchz)
names(nonmatchmean) = c("x", "y")

matchvar = totalcorset %>% filter(match == "match") %>% select(zx, zy) %>% var

nonmatchvar = totalcorset %>% filter(match == "nonmatch") %>% select(zx, zy) %>% var

########### t distribution stuff
library(mvtnorm)

nu <- 10
rtsq <- rowSums(x = matrix(rt(n = 2e6, df = nu)^2, ncol = 2))


############

lineset <- totalcorset %>% filter(knife == "T10:T10") %>% arrange(x)

newellipseplot = ggplot(mapping = aes(x=x, y=y)) +
  geom_point(data = totalcorset, mapping = aes(shape = match, color = match,
                                               alpha = I(0.9)))+
  guides(alpha=FALSE, fill = FALSE)+
    theme_bw() +
  theme(          legend.justification=c(0,1), legend.position=c(0,1),
                  legend.text =element_text(size = 11), legend.title = element_blank(),
                  legend.background = element_rect(fill = "white", color = "black"),
                  axis.text.x = element_text( angle = 45, hjust = 1 ),
                  legend.box.background = element_rect(colour = "black"),
                  legend.spacing.y = unit(0, "mm")) +
  labs(color = element_blank(), shape = element_blank(),
       x=bquote( '5-10'~mm^-1~'frequency range'),
       y=bquote('10-20'~mm^-1~'frequency range') ) +
  scale_y_continuous(trans = "fisher",breaks = c(-.15,0,.25,.5,.75,.90,.95,.99),
                     limits = ylimm) +
  scale_x_continuous(trans = "fisher", breaks = c(-.25,0,.25,.5,.75,.90,.95,.99),
```

```r
                       limits = xlimm) +
  scale_color_manual(values = c("match" = "#7F2704", "nonmatch" = "#00441B"))+
    scale_fill_manual(values = c("match" = "#7F2704", "nonmatch" = "#00441B"))+
#    geom_path(data = lineset, color = "blue") +
  NULL

######### add ellipses


for (i in seq(from = 0.10, to = 0.95, length.out = 50)) {

el.df <- data.frame(tanh((matchzdf) + ellipse(x = matchvar,
                                              t = sqrt(quantile(rtsq, probs = (i))))))
el1.df <- data.frame(tanh((nonmatchzdf)+ellipse(x = nonmatchvar,
                                              t = sqrt(quantile(rtsq, probs = (i))))))
names(el.df) <- c("x", "y")
names(el1.df) <- c("x", "y")


newellipseplot <- newellipseplot +
  geom_polygon(data=el.df, aes(x=x, y=y), fill = "#D94801", colour = NA,
               alpha = I(0.02)) +
  geom_polygon(data=el1.df, aes(x=x, y=y), fill = "#238B45", colour = NA,
               alpha = I(0.02))
}

###### add line, points back on top
newellipseplot <- newellipseplot +
  geom_point(data = totalcorset, mapping = aes(shape = match, color = match,
                                               alpha = I(0.9)))+
  NULL

#########

newellipseplot
xdens <- axis_canvas(newellipseplot, axis = 'x') +
  geom_histogram(data = totalcorset, mapping = aes(x = (x), after_stat(density),
                 fill = match, alpha = I(0.4)), position = 'identity') +
  geom_density(data = totalcorset, mapping = aes(x = (x), color = match))+
  scale_fill_manual(values = c("match" = "#D94801", "nonmatch" = "#238B45"))+
  scale_color_manual(values = c("match" = "#7F2704", "nonmatch" = "#00441B"))+
  scale_x_continuous(trans = "fisher", limits = xlimm) +
  NULL
ydens <- axis_canvas(newellipseplot, axis = 'y', coord_flip = TRUE) +
  geom_histogram(data = totalcorset, mapping = aes(x = (y), after_stat(density),
                  fill = match,  alpha = I(0.4)), position = 'identity') +
  geom_density(data = totalcorset, mapping = aes(x = (y), color = match))+
  scale_fill_manual(values = c("match" = "#D94801", "nonmatch" = "#238B45"))+
  scale_color_manual(values = c("match" = "#7F2704", "nonmatch" = "#00441B"))+
  scale_x_continuous(trans = "fisher",breaks = c(-.15,0,.25,.5,.75,.90,.95,.99),
                       limits = xlimm) +
  coord_flip() +
  NULL
```

```
  onep1 <- insert_xaxis_grob(newellipseplot, xdens, grid::unit(.2, 'null'),
                            position = "top")
  onep2 <- insert_yaxis_grob(onep1, ydens, grid::unit(.2, 'null'), position = 'right')
  return(onep2)
}


#plotting

basetip <- suppressWarnings(plot_gen("base-tip"))
```

```
## Scale for 'x' is already present. Adding another scale for 'x', which will
## replace the existing scale.

## Scale for 'x' is already present. Adding another scale for 'x', which will
## replace the existing scale.

## `stat_bin()` using `bins = 30`. Pick better value with `binwidth`.
## `stat_bin()` using `bins = 30`. Pick better value with `binwidth`.
```

```
replicabase <- suppressWarnings(plot_gen("replica-base"))
```

```
## Scale for 'x' is already present. Adding another scale for 'x', which will
## replace the existing scale.

## Scale for 'x' is already present. Adding another scale for 'x', which will
## replace the existing scale.

## `stat_bin()` using `bins = 30`. Pick better value with `binwidth`.
## `stat_bin()` using `bins = 30`. Pick better value with `binwidth`.
```

```
replicatip <- suppressWarnings(plot_gen("replica-tip"))
```

```
## Scale for 'x' is already present. Adding another scale for 'x', which will
## replace the existing scale.

## Scale for 'x' is already present. Adding another scale for 'x', which will
## replace the existing scale.

## `stat_bin()` using `bins = 30`. Pick better value with `binwidth`.
## `stat_bin()` using `bins = 30`. Pick better value with `binwidth`.
```

```
all1 <- ggarrange(basetip,replicabase,replicatip,nrow = 1)
title <-"         Base-Tip                                                    Replica of Tip-Base
all <- annotate_figure(all1, top = text_grob(title,size = 14))
all
```

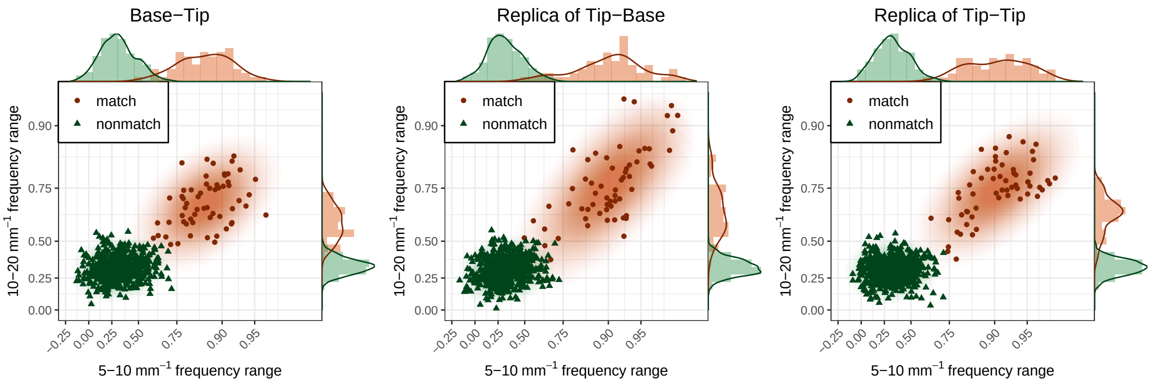


## save

```
ggsave("/home/carlos/Desktop/forensics/carlos-reports/writes/replica/codeNdata/figures/fig7/fig7.tiff",
       all, height = 4, width = 12)
```

## 2.3 Figure 8

Here we present the code that generates Figure 8. Figure 8 presents the posterior probability of match for all the cases in the log-odds scale. The classification was performed using our R package topologymatcheR, and the graph was made with the R package ggplot2.

# Figure 8

```r
library(ggplot2)
ilogit <- function(x)  exp(x)/(1+exp(x))
library(topologymatcheR)
loc <- "/home/carlos/Desktop/forensics/carlos-reports/writes/replica/codeNdata/"

#base-tip

class <- classif_gen(corrloc_train = paste0(loc,"figures/fig8/A3-corrs.csv"),
                     corrloc_test  = paste0(loc,"generating-correlations/base-tip-corrs.csv"))
```

```
## training data on n = 10 matches and n = 90 nonmatches
## testing data on n = 10 matches and n = 90 nonmatches
```

```r
base_tip <- class$probs[,1:2]
base_tip$pair <- "base-tip"


#replica-tip

class <- classif_gen(corrloc_train = paste0(loc,"figures/fig8/A3-corrs.csv"),
                     corrloc_test  = paste0(loc,"generating-correlations/replica-tip-corrs.csv"))
```

```
## training data on n = 10 matches and n = 90 nonmatches
## testing data on n = 10 matches and n = 90 nonmatches
```

```r
replica_tip <- class$probs[,1:2]
replica_tip$pair <- "replica-tip"



#replica-base

class <- classif_gen(corrloc_train = paste0(loc,"figures/fig8/A3-corrs.csv"),
                     corrloc_test  = paste0(loc,"generating-correlations/replica-base-corrs.csv"))
```

```
## training data on n = 10 matches and n = 90 nonmatches
## testing data on n = 10 matches and n = 90 nonmatches
```

```r
replica_base <- class$probs[,1:2]
replica_base$pair <- "replica-base"


allpred <- rbind(base_tip,replica_tip,replica_base)
allpred$logitprobs <- ilogit(allpred$logitprobs)

sci <- function(l){
  if(l == 0.5) return(0.5)
  # turn in to character string in scientific notation, and remove the first 1
```

```
  l <- sub('.', '', format(l, scientific = TRUE))
  # turn the 'e+' into plotmath format
  gsub("e", "10^", l)
}
fancy_scientific <- function(l) {
  l2 <- rep(0,length(l))
  for(i in 1:length(l)){
    if(l[i]<=0.5){
      l2[i] <- sci(l[i])
    } else {
      l2[i] <- paste0("1-",sci(1-l[i]))
    }
  }
  parse(text=l2)
}
ybreaks <- c(10^-(2*(4:1)),0.5,1-10^-(2*(1:4)))
ybrlab <- c("0.00000001","0.000001","0.0001","0.01","0.5","0.99","0.9999","0.99999",".99999999")
p1 <- ggplot(data = allpred, aes(x = match, y = logitprobs)) +
      labs(x = "match",y = "posterior probability of match") +
      theme_bw() +   geom_boxplot(aes(fill=pair),na.rm=TRUE) +
      geom_hline(yintercept=0.5)+
      scale_y_continuous(breaks=ybreaks,labels=fancy_scientific,
                          trans = "logit",
                         minor_breaks = ybreaks,
                         sec.axis = sec_axis(~.*1,breaks=ybreaks,
                                             labels = ybrlab))+
      theme(legend.position = c(0.72, 0.76),
            legend.background = element_rect(size=0.33, linetype="solid",colour ="black"))+
      guides(fill=guide_legend(title="surface pair"))
p1
```

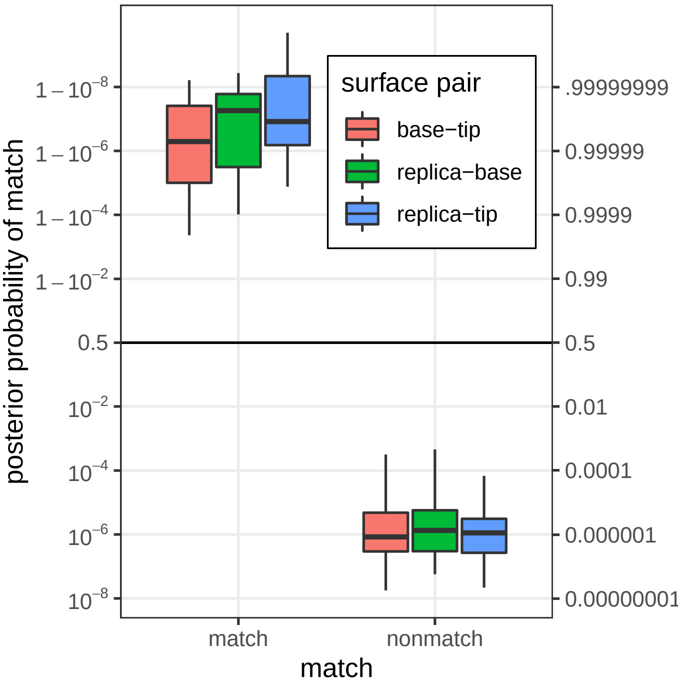


## save

```
ggsave("/home/carlos/Desktop/forensics/carlos-reports/writes/replica/codeNdata/figures/fig8/fig8.tiff",
       p1,   # The directory you want to save the file in
       width = 4, # The width of the plot in inches
       height = 4)
```

## 2.4 Figure 9

Here we present the code that generates Figure 9. Figure 9 presents mean correlations as estimated from the matrix-variate-t distribution along 10 frequency bands, for both matches and non-matches, and for the raw FFTs as well as filtered FFTs. The confidence bands were performed using parametric bootstrap with implementation based on our R package topologymatcheR. The graph was made using the R package ggplot2.

# Figure 9

```
seed <- sample(1:10000,1)
set.seed(seed)
seed
```

```
## [1] 1022
```

```
library(topologymatcheR)
library(MixMatrix)
loc <- "/home/carlos/Desktop/forensics/carlos-reports/writes/replica/codeNdata/"
B <- 500

#parametric bootstrap for 3cross-average
dat_raw <- arraycorr_gen(paste0(loc,"generating-correlations/3crossavg/3crossavg-replica-tip-corrs.csv"[illegible]
                         c(3,5,10,20,25,33,50,67,100,133,200))
mat_fit <- MLmatrixt_ar1(dat_raw$corrs[,,dat_raw$matches == "match"])
nmat_fit <- MLmatrixt_ar1(dat_raw$corrs[,,dat_raw$matches == "nonmatch"])

seeds <- sample(1:B^2)[1:B]
allMeans <- array(0,c(10,2,B))
for(i in 1:B){
 # cat("just began bootstrap #",i,"\n")
  set.seed(seeds[i])
  allMeans[,1,i] <- MLmatrixt_ar1(rmatrixt(10,5,mat_fit$mean, U=mat_fit$U, V=mat_fit$V))$mean[,1]
  allMeans[,2,i] <- MLmatrixt_ar1(rmatrixt(90,5,nmat_fit$mean, U=nmat_fit$U, V=nmat_fit$V))$mean[,1]
}
boot_3cross <- tanh(allMeans)
int_3cross <- apply(boot_3cross,1:2,function(x)quantile(x,c(0.025,0.5,0.975)))


#parametric bootstrap for raw
dat_raw <- arraycorr_gen(paste0(loc,"generating-correlations/replica-tip-corrs.csv"),
                         c(3,5,10,20,25,33,50,67,100,133,200))
mat_fit <- MLmatrixt_ar1(dat_raw$corrs[,,dat_raw$matches == "match"])
nmat_fit <- MLmatrixt_ar1(dat_raw$corrs[,,dat_raw$matches == "nonmatch"])

seeds <- sample(1:B^2)[1:B]
allMeans <- array(0,c(10,2,B))
for(i in 1:B){
 # cat("just began bootstrap #",i,"\n")
  set.seed(seeds[i])
  allMeans[,1,i] <- MLmatrixt_ar1(rmatrixt(10,5,mat_fit$mean, U=mat_fit$U, V=mat_fit$V))$mean[,1]
  allMeans[,2,i] <- MLmatrixt_ar1(rmatrixt(90,5,nmat_fit$mean, U=nmat_fit$U, V=nmat_fit$V))$mean[,1]
}
boot_raw <- tanh(allMeans)
int_raw <- apply(boot_raw,1:2,function(x)quantile(x,c(0.025,0.5,0.975)))
```

```r
#making figures

labs <- c("3-5","5-10","10-20","20-25","25-33","33-50","50-67","67-100","100-133","133-200")
dimnames(int_3cross) <- dimnames(int_raw) <- list(quantiles = c("lower","est","upper"),
                                                  bands = labs,
                                                  type = c("match","nonmatch"))
int_3cross <- data.frame(t(tensr::mat(int_3cross,1)))
int_raw <- data.frame(t(tensr::mat(int_raw,1)))

int_3cross$bands <- int_raw$bands <- rep(labs,2)
int_3cross$type <- int_raw$type <- rep(c("match","nonmatch"),each =10)

int_3cross$filter <- "3 average kernel"

int_raw$filter <- "no filter"


Alldat <- rbind(int_raw,int_3cross)

Alldat$group <- rep(1:4,each = 10)
Alldat$bands <- factor(Alldat$bands,levels=labs)
#plotting the correlations

library(ggplot2)


ybreaks <- round(tanh((0:8)/3.2+0.01),3)
p1 <- ggplot(data=Alldat, aes(x=bands, y=est , colour = type,group = group)) +
    geom_point() +
    geom_line() +
    theme_bw() +
    facet_grid(.~filter)+
    scale_y_continuous(trans = "atanh",
                       breaks=ybreaks,
                       minor_breaks = ybreaks)+
    xlab(bquote( 'frequency bands'~(mm^-1)))+
    ylab("correlation")+
    geom_ribbon(aes(ymin=lower, ymax=upper,fill=type),
                linetype=1, alpha=0.2,size=0.1)+
  ggtitle("Mean correlation")+
  theme(axis.text.x = element_text(angle = 90, vjust = 0.5, hjust=1),
        plot.title = element_text(hjust = 0.5),
        legend.title = element_blank(),
        legend.position = c(0.85, 0.75))
p1
```

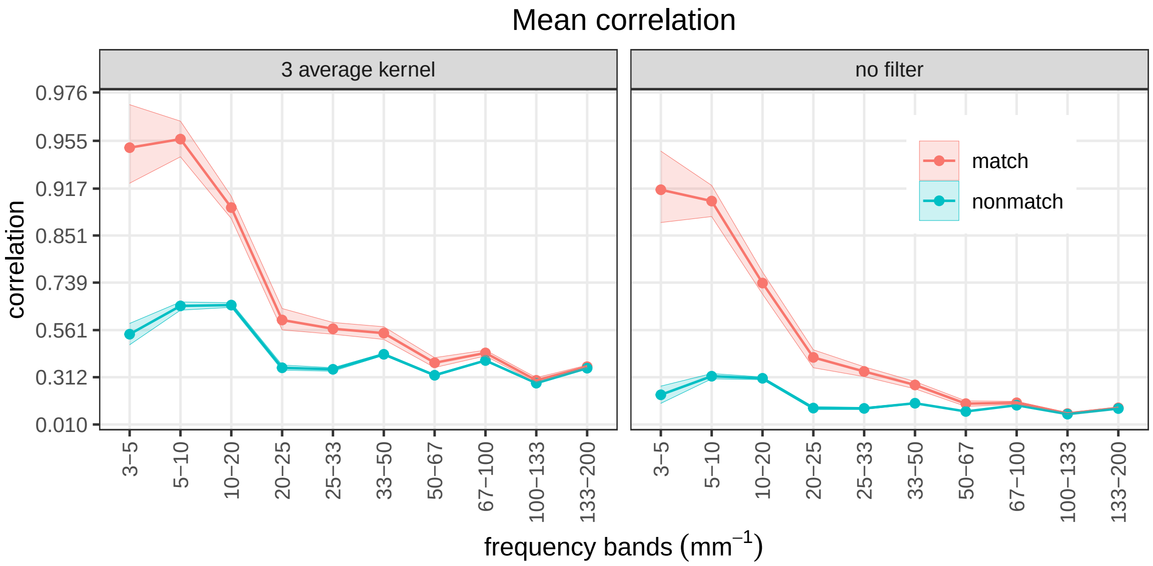


## save

```
ggsave("/home/carlos/Desktop/forensics/carlos-reports/writes/replica/codeNdata/figures/fig9/fig9.tiff",
       width = 6,
       height = 3.5)
```

# 3 Code for generating correlations

Here we present the code that was used to obtain the correlations from the raw FFTs. The correlations were obtained using our R package topologymatcheR. Section 3.1 is for obtaining the correlation that corresponds to the three pairs base-tip, base-replica, replica-tip. Section 3.2 is for obtaining the correlations that corresponds to replica-tip, but after the FFT raw data was averaged using a 3-cross filter.

### 3.1 For base-tip, replica-base and replica-tip (for Figures 6-9)

```
R version 4.1.1 (2021-08-10) -- "Kick Things"
Copyright (C) 2021 The R Foundation for Statistical Computing
Platform: x86_64-pc-linux-gnu (64-bit)

R is free software and comes with ABSOLUTELY NO WARRANTY.
You are welcome to redistribute it under certain conditions.
Type 'license()' or 'licence()' for distribution details.

  Natural language support but running in an English locale

R is a collaborative project with many contributors.
Type 'contributors()' for more information and
'citation()' on how to cite R or R packages in publications.

Type 'demo()' for some demos, 'help()' for on-line help, or
'help.start()' for an HTML browser interface to help.
Type 'q()' to quit R.

> library(topologymatcheR)
>
>
> dir_all <- "/home/carlos/Desktop/forensics/carlos-reports/writes/replica/codeNdata/"
> cuts <- c(3,5,10,20,25,33,50,67,100,133,200)
> freqs <- rbind(cbind(cuts[1:3],cuts[2:4]),
+                matrix(c(20,30,30,40),2),
+                cbind(cuts[4:10],cuts[5:11]))
> freqs
      [,1] [,2]
 [1,]    3    5
 [2,]    5   10
 [3,]   10   20
 [4,]   20   30
 [5,]   30   40
 [6,]   20   25
 [7,]   25   33
 [8,]   33   50
 [9,]   50   67
[10,]   67  100
[11,]  100  133
[12,]  133  200
> #This finds the correlations for base-tip
>
> corrs <- gencorr(img.no = 6,
+                  sample.names = c(paste0("S0",1:9),"S10"),
+                  base.names = "Abdul-5_FFT",
+                  tip.names = "Abdul-5_FFT",
+                  surface = c("Base", "Tip"),
+                  base.directory = paste0(dir_all,"FFTs/FFT-raw/"),
+                  freqs=freqs,
+                  corr_csvloc = paste0(dir_all,"generating-correlations/base-tip-corrs.csv"),
+                  corr_pdfloc = paste0(dir_all,"generating-correlations/base-tip-hist.pdf"))
dimension of first base and tip are: 512 1023
##################
 started reading data for sample S01
reading base-tip for image 1--read!
reading base-tip for image 2--read!
reading base-tip for image 3--read!
reading base-tip for image 4--read!
reading base-tip for image 5--read!
reading base-tip for image 6--read!
Finished!
```

```
##################
 started reading data for sample S02
reading base-tip for image 1--read!
reading base-tip for image 2--read!
reading base-tip for image 3--read!
reading base-tip for image 4--read!
reading base-tip for image 5--read!
reading base-tip for image 6--read!
Finished!

##################
 started reading data for sample S03
reading base-tip for image 1--read!
reading base-tip for image 2--read!
reading base-tip for image 3--read!
reading base-tip for image 4--read!
reading base-tip for image 5--read!
reading base-tip for image 6--read!
Finished!

##################
 started reading data for sample S04
reading base-tip for image 1--read!
reading base-tip for image 2--read!
reading base-tip for image 3--read!
reading base-tip for image 4--read!
reading base-tip for image 5--read!
reading base-tip for image 6--read!
Finished!

##################
 started reading data for sample S05
reading base-tip for image 1--read!
reading base-tip for image 2--read!
reading base-tip for image 3--read!
reading base-tip for image 4--read!
reading base-tip for image 5--read!
reading base-tip for image 6--read!
Finished!

##################
 started reading data for sample S06
reading base-tip for image 1--read!
reading base-tip for image 2--read!
reading base-tip for image 3--read!
reading base-tip for image 4--read!
reading base-tip for image 5--read!
reading base-tip for image 6--read!
Finished!

##################
 started reading data for sample S07
reading base-tip for image 1--read!
reading base-tip for image 2--read!
reading base-tip for image 3--read!
reading base-tip for image 4--read!
reading base-tip for image 5--read!
reading base-tip for image 6--read!
Finished!

##################
 started reading data for sample S08
reading base-tip for image 1--read!
```

```
reading base-tip for image 2--read!
reading base-tip for image 3--read!
reading base-tip for image 4--read!
reading base-tip for image 5--read!
reading base-tip for image 6--read!
Finished!

##################
 started reading data for sample S09
reading base-tip for image 1--read!
reading base-tip for image 2--read!
reading base-tip for image 3--read!
reading base-tip for image 4--read!
reading base-tip for image 5--read!
reading base-tip for image 6--read!
Finished!

##################
 started reading data for sample S10
reading base-tip for image 1--read!
reading base-tip for image 2--read!
reading base-tip for image 3--read!
reading base-tip for image 4--read!
reading base-tip for image 5--read!
reading base-tip for image 6--read!
Finished!

Finished reading data, now creating correlations
Finding correlations along the frequency bands  3-5 5-10 10-20 20-30 30-40 20-25 25-33 33-50 5…
generating correlations along the frequency band  3-5 : Finished: Matches and Non-Matches
generating correlations along the frequency band  5-10 : Finished: Matches and Non-Matches
generating correlations along the frequency band  10-20 : Finished: Matches and Non-Matches
generating correlations along the frequency band  20-30 : Finished: Matches and Non-Matches
generating correlations along the frequency band  30-40 : Finished: Matches and Non-Matches
generating correlations along the frequency band  20-25 : Finished: Matches and Non-Matches
generating correlations along the frequency band  25-33 : Finished: Matches and Non-Matches
generating correlations along the frequency band  33-50 : Finished: Matches and Non-Matches
generating correlations along the frequency band  50-67 : Finished: Matches and Non-Matches
generating correlations along the frequency band  67-100 : Finished: Matches and Non-Matches
generating correlations along the frequency band  100-133 : Finished: Matches and Non-Matches
generating correlations along the frequency band  133-200 : Finished: Matches and Non-Matches
Finished creating correlations!
Finished saving csv correlation file
Finished saving pdf correlation file
>
>
> #This finds the correlations for replica-base
>
> corrs <- gencorr(img.no = 6,
+                  sample.names = c(paste0("S0",1:9),"S10"),
+                  base.names = "Abdul-5_FFT",
+                  tip.names = "Abdul-Rep_FFT",
+                  surface = c("Base", "Base"),
+                  base.directory = paste0(dir_all,"FFTs/FFT-raw/"),
+                  freqs=freqs,
+                  corr_csvloc = paste0(dir_all,"generating-correlations/replica-base-corrs.cs…
+                  corr_pdfloc = paste0(dir_all,"generating-correlations/replica-base-hist.pdf…
dimension of first base and tip are: 512 1023
##################
 started reading data for sample S01
reading base-tip for image 1--read!
reading base-tip for image 2--read!
reading base-tip for image 3--read!
```

```
reading base-tip for image 4--read!
reading base-tip for image 5--read!
reading base-tip for image 6--read!
Finished!

##################
 started reading data for sample S02
reading base-tip for image 1--read!
reading base-tip for image 2--read!
reading base-tip for image 3--read!
reading base-tip for image 4--read!
reading base-tip for image 5--read!
reading base-tip for image 6--read!
Finished!

##################
 started reading data for sample S03
reading base-tip for image 1--read!
reading base-tip for image 2--read!
reading base-tip for image 3--read!
reading base-tip for image 4--read!
reading base-tip for image 5--read!
reading base-tip for image 6--read!
Finished!

##################
 started reading data for sample S04
reading base-tip for image 1--read!
reading base-tip for image 2--read!
reading base-tip for image 3--read!
reading base-tip for image 4--read!
reading base-tip for image 5--read!
reading base-tip for image 6--read!
Finished!

##################
 started reading data for sample S05
reading base-tip for image 1--read!
reading base-tip for image 2--read!
reading base-tip for image 3--read!
reading base-tip for image 4--read!
reading base-tip for image 5--read!
reading base-tip for image 6--read!
Finished!

##################
 started reading data for sample S06
reading base-tip for image 1--read!
reading base-tip for image 2--read!
reading base-tip for image 3--read!
reading base-tip for image 4--read!
reading base-tip for image 5--read!
reading base-tip for image 6--read!
Finished!

##################
 started reading data for sample S07
reading base-tip for image 1--read!
reading base-tip for image 2--read!
reading base-tip for image 3--read!
reading base-tip for image 4--read!
reading base-tip for image 5--read!
reading base-tip for image 6--read!
```

```
Finished!

##################
 started reading data for sample S08
reading base-tip for image 1--read!
reading base-tip for image 2--read!
reading base-tip for image 3--read!
reading base-tip for image 4--read!
reading base-tip for image 5--read!
reading base-tip for image 6--read!
Finished!

##################
 started reading data for sample S09
reading base-tip for image 1--read!
reading base-tip for image 2--read!
reading base-tip for image 3--read!
reading base-tip for image 4--read!
reading base-tip for image 5--read!
reading base-tip for image 6--read!
Finished!

##################
 started reading data for sample S10
reading base-tip for image 1--read!
reading base-tip for image 2--read!
reading base-tip for image 3--read!
reading base-tip for image 4--read!
reading base-tip for image 5--read!
reading base-tip for image 6--read!
Finished!

Finished reading data, now creating correlations
Finding correlations along the frequency bands  3-5 5-10 10-20 20-30 30-40 20-25 25-33 33-50 5…
generating correlations along the frequency band  3-5 : Finished: Matches and Non-Matches
generating correlations along the frequency band  5-10 : Finished: Matches and Non-Matches
generating correlations along the frequency band  10-20 : Finished: Matches and Non-Matches
generating correlations along the frequency band  20-30 : Finished: Matches and Non-Matches
generating correlations along the frequency band  30-40 : Finished: Matches and Non-Matches
generating correlations along the frequency band  20-25 : Finished: Matches and Non-Matches
generating correlations along the frequency band  25-33 : Finished: Matches and Non-Matches
generating correlations along the frequency band  33-50 : Finished: Matches and Non-Matches
generating correlations along the frequency band  50-67 : Finished: Matches and Non-Matches
generating correlations along the frequency band  67-100 : Finished: Matches and Non-Matches
generating correlations along the frequency band  100-133 : Finished: Matches and Non-Matches
generating correlations along the frequency band  133-200 : Finished: Matches and Non-Matches
Finished creating correlations!
Finished saving csv correlation file
Finished saving pdf correlation file
>
>
>
> #This finds the correlations for replica-tip
>
> corrs <- gencorr(img.no = 6,
+                  sample.names = c(paste0("S0",1:9),"S10"),
+                  base.names = "Abdul-Rep_FFT",
+                  tip.names = "Abdul-5_FFT",
+                  surface = c("Base", "Tip"),
+                  base.directory = paste0(dir_all,"FFTs/FFT-raw/"),
+                  freqs=freqs,
+                  corr_csvloc = paste0(dir_all,"generating-correlations/replica-tip-corrs.csv…
+                  corr_pdfloc = paste0(dir_all,"generating-correlations/replica-tip-hist.pdf"…
```

```
dimension of first base and tip are: 512 1023
##################
 started reading data for sample S01
reading base-tip for image 1--read!
reading base-tip for image 2--read!
reading base-tip for image 3--read!
reading base-tip for image 4--read!
reading base-tip for image 5--read!
reading base-tip for image 6--read!
Finished!

##################
 started reading data for sample S02
reading base-tip for image 1--read!
reading base-tip for image 2--read!
reading base-tip for image 3--read!
reading base-tip for image 4--read!
reading base-tip for image 5--read!
reading base-tip for image 6--read!
Finished!

##################
 started reading data for sample S03
reading base-tip for image 1--read!
reading base-tip for image 2--read!
reading base-tip for image 3--read!
reading base-tip for image 4--read!
reading base-tip for image 5--read!
reading base-tip for image 6--read!
Finished!

##################
 started reading data for sample S04
reading base-tip for image 1--read!
reading base-tip for image 2--read!
reading base-tip for image 3--read!
reading base-tip for image 4--read!
reading base-tip for image 5--read!
reading base-tip for image 6--read!
Finished!

##################
 started reading data for sample S05
reading base-tip for image 1--read!
reading base-tip for image 2--read!
reading base-tip for image 3--read!
reading base-tip for image 4--read!
reading base-tip for image 5--read!
reading base-tip for image 6--read!
Finished!

##################
 started reading data for sample S06
reading base-tip for image 1--read!
reading base-tip for image 2--read!
reading base-tip for image 3--read!
reading base-tip for image 4--read!
reading base-tip for image 5--read!
reading base-tip for image 6--read!
Finished!

##################
 started reading data for sample S07
```

```
reading base-tip for image 1--read!
reading base-tip for image 2--read!
reading base-tip for image 3--read!
reading base-tip for image 4--read!
reading base-tip for image 5--read!
reading base-tip for image 6--read!
Finished!

##################
 started reading data for sample S08
reading base-tip for image 1--read!
reading base-tip for image 2--read!
reading base-tip for image 3--read!
reading base-tip for image 4--read!
reading base-tip for image 5--read!
reading base-tip for image 6--read!
Finished!

##################
 started reading data for sample S09
reading base-tip for image 1--read!
reading base-tip for image 2--read!
reading base-tip for image 3--read!
reading base-tip for image 4--read!
reading base-tip for image 5--read!
reading base-tip for image 6--read!
Finished!

##################
 started reading data for sample S10
reading base-tip for image 1--read!
reading base-tip for image 2--read!
reading base-tip for image 3--read!
reading base-tip for image 4--read!
reading base-tip for image 5--read!
reading base-tip for image 6--read!
Finished!

Finished reading data, now creating correlations
Finding correlations along the frequency bands  3-5 5-10 10-20 20-30 30-40 20-25 25-33 33-50 5…
generating correlations along the frequency band  3-5 : Finished: Matches and Non-Matches
generating correlations along the frequency band  5-10 : Finished: Matches and Non-Matches
generating correlations along the frequency band  10-20 : Finished: Matches and Non-Matches
generating correlations along the frequency band  20-30 : Finished: Matches and Non-Matches
generating correlations along the frequency band  30-40 : Finished: Matches and Non-Matches
generating correlations along the frequency band  20-25 : Finished: Matches and Non-Matches
generating correlations along the frequency band  25-33 : Finished: Matches and Non-Matches
generating correlations along the frequency band  33-50 : Finished: Matches and Non-Matches
generating correlations along the frequency band  50-67 : Finished: Matches and Non-Matches
generating correlations along the frequency band  67-100 : Finished: Matches and Non-Matches
generating correlations along the frequency band  100-133 : Finished: Matches and Non-Matches
generating correlations along the frequency band  133-200 : Finished: Matches and Non-Matches
Finished creating correlations!
Finished saving csv correlation file
Finished saving pdf correlation file
>
> q('no')
> proc.time()
    user   system  elapsed
1359.816   68.692 1441.433
```

### 3.2 For averaged replica-tip (for Figure 9)

```
R version 4.1.1 (2021-08-10) -- "Kick Things"
Copyright (C) 2021 The R Foundation for Statistical Computing
Platform: x86_64-pc-linux-gnu (64-bit)

R is free software and comes with ABSOLUTELY NO WARRANTY.
You are welcome to redistribute it under certain conditions.
Type 'license()' or 'licence()' for distribution details.

  Natural language support but running in an English locale

R is a collaborative project with many contributors.
Type 'contributors()' for more information and
'citation()' on how to cite R or R packages in publications.

Type 'demo()' for some demos, 'help()' for on-line help, or
'help.start()' for an HTML browser interface to help.
Type 'q()' to quit R.

> library(topologymatcheR)
>
>
> dir_all <- "/home/carlos/Desktop/forensics/carlos-reports/writes/replica/codeNdata/"
> cuts <- c(3,5,10,20,25,33,50,67,100,133,200)
> freqs <- rbind(cbind(cuts[1:3],cuts[2:4]),
+                matrix(c(20,30,30,40),2),
+                cbind(cuts[4:10],cuts[5:11]))
> freqs
      [,1] [,2]
 [1,]    3    5
 [2,]    5   10
 [3,]   10   20
 [4,]   20   30
 [5,]   30   40
 [6,]   20   25
 [7,]   25   33
 [8,]   33   50
 [9,]   50   67
[10,]   67  100
[11,]  100  133
[12,]  133  200
>
>
> #This finds the correlations for replica-tip
>
> corrs <- gencorr(img.no = 6,
+                  sample.names = c(paste0("S0",1:9),"S10"),
+                  base.names = "Abdul-Rep_FFT",
+                  tip.names = "Abdul-5_FFT",
+                  surface = c("Base", "Tip"),
+                  base.directory = paste0(dir_all,"FFTs/FFT-3crossaveraged/"),
+                  freqs=freqs,
+                  corr_csvloc = paste0(dir_all,"generating-correlations/3crossavg/3crossavg-r…
+                  corr_pdfloc = paste0(dir_all,"generating-correlations/3crossavg/3crossavg-r…
dimension of first base and tip are: 512 1023
##################
 started reading data for sample S01
reading base-tip for image 1--read!
reading base-tip for image 2--read!
reading base-tip for image 3--read!
reading base-tip for image 4--read!
reading base-tip for image 5--read!
reading base-tip for image 6--read!
```

```
Finished!

##################
 started reading data for sample S02
reading base-tip for image 1--read!
reading base-tip for image 2--read!
reading base-tip for image 3--read!
reading base-tip for image 4--read!
reading base-tip for image 5--read!
reading base-tip for image 6--read!
Finished!

##################
 started reading data for sample S03
reading base-tip for image 1--read!
reading base-tip for image 2--read!
reading base-tip for image 3--read!
reading base-tip for image 4--read!
reading base-tip for image 5--read!
reading base-tip for image 6--read!
Finished!

##################
 started reading data for sample S04
reading base-tip for image 1--read!
reading base-tip for image 2--read!
reading base-tip for image 3--read!
reading base-tip for image 4--read!
reading base-tip for image 5--read!
reading base-tip for image 6--read!
Finished!

##################
 started reading data for sample S05
reading base-tip for image 1--read!
reading base-tip for image 2--read!
reading base-tip for image 3--read!
reading base-tip for image 4--read!
reading base-tip for image 5--read!
reading base-tip for image 6--read!
Finished!

##################
 started reading data for sample S06
reading base-tip for image 1--read!
reading base-tip for image 2--read!
reading base-tip for image 3--read!
reading base-tip for image 4--read!
reading base-tip for image 5--read!
reading base-tip for image 6--read!
Finished!

##################
 started reading data for sample S07
reading base-tip for image 1--read!
reading base-tip for image 2--read!
reading base-tip for image 3--read!
reading base-tip for image 4--read!
reading base-tip for image 5--read!
reading base-tip for image 6--read!
Finished!

##################
```

```
 started reading data for sample S08
reading base-tip for image 1--read!
reading base-tip for image 2--read!
reading base-tip for image 3--read!
reading base-tip for image 4--read!
reading base-tip for image 5--read!
reading base-tip for image 6--read!
Finished!

##################
 started reading data for sample S09
reading base-tip for image 1--read!
reading base-tip for image 2--read!
reading base-tip for image 3--read!
reading base-tip for image 4--read!
reading base-tip for image 5--read!
reading base-tip for image 6--read!
Finished!

##################
 started reading data for sample S10
reading base-tip for image 1--read!
reading base-tip for image 2--read!
reading base-tip for image 3--read!
reading base-tip for image 4--read!
reading base-tip for image 5--read!
reading base-tip for image 6--read!
Finished!

Finished reading data, now creating correlations
Finding correlations along the frequency bands  3-5 5-10 10-20 20-30 30-40 20-25 25-33 33-50 5…
generating correlations along the frequency band  3-5 : Finished: Matches and Non-Matches
generating correlations along the frequency band  5-10 : Finished: Matches and Non-Matches
generating correlations along the frequency band  10-20 : Finished: Matches and Non-Matches
generating correlations along the frequency band  20-30 : Finished: Matches and Non-Matches
generating correlations along the frequency band  30-40 : Finished: Matches and Non-Matches
generating correlations along the frequency band  20-25 : Finished: Matches and Non-Matches
generating correlations along the frequency band  25-33 : Finished: Matches and Non-Matches
generating correlations along the frequency band  33-50 : Finished: Matches and Non-Matches
generating correlations along the frequency band  50-67 : Finished: Matches and Non-Matches
generating correlations along the frequency band  67-100 : Finished: Matches and Non-Matches
generating correlations along the frequency band  100-133 : Finished: Matches and Non-Matches
generating correlations along the frequency band  133-200 : Finished: Matches and Non-Matches
Finished creating correlations!
Finished saving csv correlation file
Finished saving pdf correlation file
>
> q('no')
> proc.time()
   user  system elapsed
429.191  32.167 463.969
```

# 4 3-cross averaging of raw FFT data

This code performs the 3-cross averaging of FFTs. The R packages readxl and writexl are used.

```
R version 4.1.1 (2021-08-10) -- "Kick Things"
Copyright (C) 2021 The R Foundation for Statistical Computing
Platform: x86_64-pc-linux-gnu (64-bit)

R is free software and comes with ABSOLUTELY NO WARRANTY.
You are welcome to redistribute it under certain conditions.
Type 'license()' or 'licence()' for distribution details.

  Natural language support but running in an English locale

R is a collaborative project with many contributors.
Type 'contributors()' for more information and
'citation()' on how to cite R or R packages in publications.

Type 'demo()' for some demos, 'help()' for on-line help, or
'help.start()' for an HTML browser interface to help.
Type 'q()' to quit R.

> library(writexl)
> library(readxl)
> library(foreach)
> library(doParallel)
Loading required package: iterators
Loading required package: parallel
> orig <- "/home/carlos/Desktop/forensics/carlos-reports/writes/replica/codeNdata/FFTs/FFT-raw…
> targ <- "/home/carlos/Desktop/forensics/carlos-reports/writes/replica/codeNdata/FFTs/FFT-3cr…
> fils <- list.files(orig)
> fils <- fils[!(substr(fils,19,19) == "5" & substr(fils,5,5) == "B")]
>
> inds <- function(i,j,right=F,bottom=F,rb = F){
+   d1 <- cbind((i-1):(i+1),j)
+   d2 <- cbind(i,c(j-1,j+1))
+   d3 <- rbind(d1,d2)
+   if(right) return(d3[1:4,])
+   if(bottom) return(d3[c(1:2,4:5),])
+   if(rb) return(d3[c(1,2,4),])
+   d3
+ }
>
>
> for(k in 1:length(fils)){
+   cat("performing :    ",fils[k],"\n")
+   mat.raw <- as.matrix(read_excel(path = paste0(orig,fils[k]), col_names = FALSE))
+   mat.new <- mat.raw
+   for(j in 2:ncol(mat.raw)) for(i in 2:nrow(mat.raw)){
+     if(i == 512 && j == 1023){
+       ind <- inds(i,j,rb=T)
+     } else if (i == 512){
+       ind <- inds(i,j,bottom=T)
+     } else if (j == 1023){
+       ind <- inds(i,j,right=T)
+     } else {
+       ind <- inds(i,j)
+     }
+     mat.new[i,j] <- mean(mat.raw[ind])
+   }
+   mat.new <- data.frame(mat.new)
+   write_xlsx(mat.new, paste0(targ,fils[k]),col_names = FALSE)
+ }
performing :     S01-Base-01-Abdul-Rep_FFT.xlsx
performing :     S01-Base-02-Abdul-Rep_FFT.xlsx
performing :     S01-Base-03-Abdul-Rep_FFT.xlsx
```

```
performing :    S01-Base-04-Abdul-Rep_FFT.xlsx
performing :    S01-Base-05-Abdul-Rep_FFT.xlsx
performing :    S01-Base-06-Abdul-Rep_FFT.xlsx
performing :    S01-Tip-01-Abdul-5_FFT.xlsx
performing :    S01-Tip-02-Abdul-5_FFT.xlsx
performing :    S01-Tip-03-Abdul-5_FFT.xlsx
performing :    S01-Tip-04-Abdul-5_FFT.xlsx
performing :    S01-Tip-05-Abdul-5_FFT.xlsx
performing :    S01-Tip-06-Abdul-5_FFT.xlsx
performing :    S02-Base-01-Abdul-Rep_FFT.xlsx
performing :    S02-Base-02-Abdul-Rep_FFT.xlsx
performing :    S02-Base-03-Abdul-Rep_FFT.xlsx
performing :    S02-Base-04-Abdul-Rep_FFT.xlsx
performing :    S02-Base-05-Abdul-Rep_FFT.xlsx
performing :    S02-Base-06-Abdul-Rep_FFT.xlsx
performing :    S02-Tip-01-Abdul-5_FFT.xlsx
performing :    S02-Tip-02-Abdul-5_FFT.xlsx
performing :    S02-Tip-03-Abdul-5_FFT.xlsx
performing :    S02-Tip-04-Abdul-5_FFT.xlsx
performing :    S02-Tip-05-Abdul-5_FFT.xlsx
performing :    S02-Tip-06-Abdul-5_FFT.xlsx
performing :    S03-Base-01-Abdul-Rep_FFT.xlsx
performing :    S03-Base-02-Abdul-Rep_FFT.xlsx
performing :    S03-Base-03-Abdul-Rep_FFT.xlsx
performing :    S03-Base-04-Abdul-Rep_FFT.xlsx
performing :    S03-Base-05-Abdul-Rep_FFT.xlsx
performing :    S03-Base-06-Abdul-Rep_FFT.xlsx
performing :    S03-Tip-01-Abdul-5_FFT.xlsx
performing :    S03-Tip-02-Abdul-5_FFT.xlsx
performing :    S03-Tip-03-Abdul-5_FFT.xlsx
performing :    S03-Tip-04-Abdul-5_FFT.xlsx
performing :    S03-Tip-05-Abdul-5_FFT.xlsx
performing :    S03-Tip-06-Abdul-5_FFT.xlsx
performing :    S04-Base-01-Abdul-Rep_FFT.xlsx
performing :    S04-Base-02-Abdul-Rep_FFT.xlsx
performing :    S04-Base-03-Abdul-Rep_FFT.xlsx
performing :    S04-Base-04-Abdul-Rep_FFT.xlsx
performing :    S04-Base-05-Abdul-Rep_FFT.xlsx
performing :    S04-Base-06-Abdul-Rep_FFT.xlsx
performing :    S04-Tip-01-Abdul-5_FFT.xlsx
performing :    S04-Tip-02-Abdul-5_FFT.xlsx
performing :    S04-Tip-03-Abdul-5_FFT.xlsx
performing :    S04-Tip-04-Abdul-5_FFT.xlsx
performing :    S04-Tip-05-Abdul-5_FFT.xlsx
performing :    S04-Tip-06-Abdul-5_FFT.xlsx
performing :    S05-Base-01-Abdul-Rep_FFT.xlsx
performing :    S05-Base-02-Abdul-Rep_FFT.xlsx
performing :    S05-Base-03-Abdul-Rep_FFT.xlsx
performing :    S05-Base-04-Abdul-Rep_FFT.xlsx
performing :    S05-Base-05-Abdul-Rep_FFT.xlsx
performing :    S05-Base-06-Abdul-Rep_FFT.xlsx
performing :    S05-Tip-01-Abdul-5_FFT.xlsx
performing :    S05-Tip-02-Abdul-5_FFT.xlsx
performing :    S05-Tip-03-Abdul-5_FFT.xlsx
performing :    S05-Tip-04-Abdul-5_FFT.xlsx
performing :    S05-Tip-05-Abdul-5_FFT.xlsx
performing :    S05-Tip-06-Abdul-5_FFT.xlsx
performing :    S06-Base-01-Abdul-Rep_FFT.xlsx
performing :    S06-Base-02-Abdul-Rep_FFT.xlsx
performing :    S06-Base-03-Abdul-Rep_FFT.xlsx
performing :    S06-Base-04-Abdul-Rep_FFT.xlsx
performing :    S06-Base-05-Abdul-Rep_FFT.xlsx
performing :    S06-Base-06-Abdul-Rep_FFT.xlsx
```

```
performing :    S06-Tip-01-Abdul-5_FFT.xlsx
performing :    S06-Tip-02-Abdul-5_FFT.xlsx
performing :    S06-Tip-03-Abdul-5_FFT.xlsx
performing :    S06-Tip-04-Abdul-5_FFT.xlsx
performing :    S06-Tip-05-Abdul-5_FFT.xlsx
performing :    S06-Tip-06-Abdul-5_FFT.xlsx
performing :    S07-Base-01-Abdul-Rep_FFT.xlsx
performing :    S07-Base-02-Abdul-Rep_FFT.xlsx
performing :    S07-Base-03-Abdul-Rep_FFT.xlsx
performing :    S07-Base-04-Abdul-Rep_FFT.xlsx
performing :    S07-Base-05-Abdul-Rep_FFT.xlsx
performing :    S07-Base-06-Abdul-Rep_FFT.xlsx
performing :    S07-Tip-01-Abdul-5_FFT.xlsx
performing :    S07-Tip-02-Abdul-5_FFT.xlsx
performing :    S07-Tip-03-Abdul-5_FFT.xlsx
performing :    S07-Tip-04-Abdul-5_FFT.xlsx
performing :    S07-Tip-05-Abdul-5_FFT.xlsx
performing :    S07-Tip-06-Abdul-5_FFT.xlsx
performing :    S08-Base-01-Abdul-Rep_FFT.xlsx
performing :    S08-Base-02-Abdul-Rep_FFT.xlsx
performing :    S08-Base-03-Abdul-Rep_FFT.xlsx
performing :    S08-Base-04-Abdul-Rep_FFT.xlsx
performing :    S08-Base-05-Abdul-Rep_FFT.xlsx
performing :    S08-Base-06-Abdul-Rep_FFT.xlsx
performing :    S08-Tip-01-Abdul-5_FFT.xlsx
performing :    S08-Tip-02-Abdul-5_FFT.xlsx
performing :    S08-Tip-03-Abdul-5_FFT.xlsx
performing :    S08-Tip-04-Abdul-5_FFT.xlsx
performing :    S08-Tip-05-Abdul-5_FFT.xlsx
performing :    S08-Tip-06-Abdul-5_FFT.xlsx
performing :    S09-Base-01-Abdul-Rep_FFT.xlsx
performing :    S09-Base-02-Abdul-Rep_FFT.xlsx
performing :    S09-Base-03-Abdul-Rep_FFT.xlsx
performing :    S09-Base-04-Abdul-Rep_FFT.xlsx
performing :    S09-Base-05-Abdul-Rep_FFT.xlsx
performing :    S09-Base-06-Abdul-Rep_FFT.xlsx
performing :    S09-Tip-01-Abdul-5_FFT.xlsx
performing :    S09-Tip-02-Abdul-5_FFT.xlsx
performing :    S09-Tip-03-Abdul-5_FFT.xlsx
performing :    S09-Tip-04-Abdul-5_FFT.xlsx
performing :    S09-Tip-05-Abdul-5_FFT.xlsx
performing :    S09-Tip-06-Abdul-5_FFT.xlsx
performing :    S10-Base-01-Abdul-Rep_FFT.xlsx
performing :    S10-Base-02-Abdul-Rep_FFT.xlsx
performing :    S10-Base-03-Abdul-Rep_FFT.xlsx
performing :    S10-Base-04-Abdul-Rep_FFT.xlsx
performing :    S10-Base-05-Abdul-Rep_FFT.xlsx
performing :    S10-Base-06-Abdul-Rep_FFT.xlsx
performing :    S10-Tip-01-Abdul-5_FFT.xlsx
performing :    S10-Tip-02-Abdul-5_FFT.xlsx
performing :    S10-Tip-03-Abdul-5_FFT.xlsx
performing :    S10-Tip-04-Abdul-5_FFT.xlsx
performing :    S10-Tip-05-Abdul-5_FFT.xlsx
performing :    S10-Tip-06-Abdul-5_FFT.xlsx
> q('no')
> proc.time()
    user   system  elapsed
 986.396    7.383 1000.393
```
